# Web portal for analytical validation of MRM-MS assay abided with integrative multinational guidelines

**DOI:** 10.1038/s41598-020-67731-x

**Published:** 2020-07-02

**Authors:** Jaenyeon Kim, Injoon Yeo, Hyunsoo Kim, Areum Sohn, Yoseop Kim, Youngsoo Kim

**Affiliations:** 1 Interdisciplinary Program of Bioengineering, Seoul National University College of Engineering, Seoul, South Korea; 2 Institute of Medical and Biological Engineering, MRC, Seoul National University, Seoul, South Korea; 3 Department of Biomedical Sciences, Seoul National University College of Medicine, Seoul, South Korea; 4 Department of Biomedical Engineering, Seoul National University College of Medicine, Seoul, South Korea

**Keywords:** Computational biology and bioinformatics, Biomarkers

## Abstract

Multiple reaction monitoring-mass spectrometry became a mainstream method for quantitative proteomics, which made the validation of a method and the analyzed data important. In this portal for validation of the MRM-MS assay, we developed a website that automatically evaluates uploaded MRM-MS data, based on biomarker assay guidelines from the European Medicines Agency, the US Food & Drug Administration, and the Korea Food & Drug Administration. The portal reads a Skyline output file and produces the following results—calibration curve, specificity, sensitivity, carryover, precision, recovery, matrix effect, recovery, dilution integrity, stability, and QC—according to the standards of each independent agency. The final tables and figures that pertain to the 11 evaluation categories are displayed in an individual page. Spring boot was used as a framework for development of the webpage, which follows MVC Pattern. JSP, HTML, XML, and Java Script were used to develop the webpage. A server was composed of Apache Tomcat, MySQL. Input files were skyline-derived output files (csv file), and each files were organized by specific columns in order. SQL, JAVA were interworked to evaluate all the categories and show the results. Method Validation Portal can be accessed via any kind of explorer from https://pnbvalid.snu.ac.kr.

## Introduction

Biomarkers are instrumental in the detection and management of diseases^1^. Despite their many publications, novel technologies, and abundant funding, few biomarkers make it to clinical practice. This phenomenon can be partially attributed to the lack of a clear and accessible path for validating biomarker candidates for clinical use^2^.

Because biomarkers vary in their characteristics and are evaluated accordingly, it is necessary to validate biomarker assays using several criteria and methods^3,4^. For instance, blood protein-based biomarkers are often detected using quantitative immunoassays. In contrast, protein-based biomarkers and DNA-based biomarkers in tissue are generally measured using immunohistochemical and in situ hybridization assays, respectively^5^. In the past decade, mRNA-based biomarkers have been studied using microarrays^6^.

Regardless of type, a biomarker assay must be validated analytically prior to clinical use. Analytical method validation involves confirming the accuracy, precision, specificity, robustness, and stability of the biomarker assay and overall method^3,7–10^. Other assay validation criteria include linearity, parallelism, recovery following analyte addition, and functional sensitivity.

Multiple reaction monitoring-mass spectrometry (MRM-MS) assays are suitable for measuring multi-marker panels in clinical applications^11–14^. An MRM-MS assay can accurately quantify multiple biomarkers. However, the analytical validation of MRM-MS assays, which simultaneously measure thousands of transitions that correspond to quantitative values of multimarkers, can be difficult and laborious, especially if the interpretation and evaluation of numerous procedures and categories are performed manually.

Currently, MRM-MS data can be processed in part using vendor-specific software (e.g., MassHunter Quantitative Analysis, Agilent; MultiQuant, ABSciex; Pinpoint, Thermo Scientific) or vendor-independent programs, such as Skyline^15,16^. Overall, these software programs are generally used to perform a preliminary analysis of mass spectral data and transitions and also allow the user to verify and edit peak selection/integration. Qualis-SIS^17^, is a software program that was developed to automatically generate peptide standard curves and calculates assay attributes from MRM data, such as limit of quantification and dynamic range, that adhere to US Food and Drug Administration (FDA) guidelines. Although it provides intuitive results for several criteria, but it does not apply to all categories in FDA guidelines, nor to those of the EMA or KFDA. None of these software programs possesses a feature that provides insight into the analytical validation of the detected transitions in a given MRM-MS assay.

**Figure 1:**
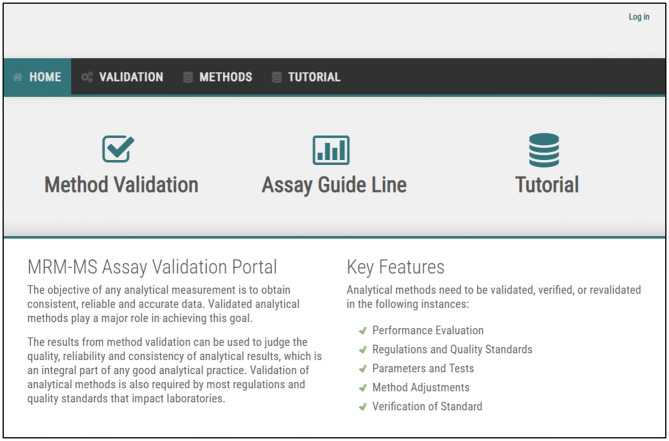
Snapshots of the M-MVP homepage for simple and advanced analytical method validation of MRM-MS assays.

The MRM-MS assay requires analytical validation to measure single or multiple biomarkers in clinical settings. However, limitations in the analytical validation of the MRM-MS assay will be encountered. To address this challenge, we developed and launched an assay portal, named M-MVP (MRM–MS assay-analytical method Validation Portal, https://pnbvalid.snu.ac.kr), as a free tool (Fig. 1). M-MVP is designed to automatically evaluate MRM-MS assay data. The method validation items configured in M-MVP are designed to meet the requirements of three sets of guidelines [US Food and Drug Administration (FDA), European Medicines Agency (EMA), and Korea Food and Drug Administration (KFDA)].

Various analytical validation procedures can be evaluated using M-MVP with minimal effort. M-MVP centralizes all method validation calculations, which significantly reduces the time, effort, and errors that would likely occur with manual processing. These advantages facilitate the implementation of MRM-MS assays in clinical settings by simplifying the analytical validation of multi-marker panel assays.

## Result

The main objective of method validation is to test the reliability of a method that is presented by the researcher for determining the concentration of 1 or more analytes in a specific biological matrix. For the method to be considered reproducible and reliable, the FDA, EMA and kFDA have established 11 criteria—some of which have been implemented across administrations—that must be fulfilled for validation. The categories are: Calibration Curve, Specificity, Sensitivity, Carryover, Precision, Accuracy, Matrix Effects, Recovery, Dilution Integrity, Stability, and Quality Control (QC).

Calibration Curve needs to be examined to show the linearity of the quantitative range of the assay that can be expected in the study. Specificity of the method requires that the target analyte and internal standards be distinguishable from endogenous components in the matrix with confidence. The lower limit of quantification (LLOQ) of the calibration curve for each analyte defines the sensitivity of the method. Validating Carryover ensures that if a high concentration of analyte in a matrix is measured, the data of the following batch will not be affected by it. Precision and Accuracy, which are self-explanatory, validate the method by assessing the closeness of repeated individual measures of analytes and the closeness of the observed value to the nominal value. Matrix effects are crucial factors to consider when a method is performed by LC–MS, because ion suppression or enhancement can occur during the experiment and impact the results. Recovery of the method requires optimization to ensure that the extraction of analyte is efficient and reproducible. Dilution Integrity ensures that diluting the matrix or the analyte does not affect the accuracy or precision. Stability determines that the analyte in the matrix is stable during handling and storage. Finally, QC evaluates the performance of the method and the stability of the target analyte.

The methods and assays are detailed in our previous study^18^ and supplementary information. For every category, regulatory agencies have specified conditions for passing the standard, and the portal automatically calculates required values from the uploaded data that correspond to each category and presents the calculated values and evaluation results on individual pages.

**Figure 2:**
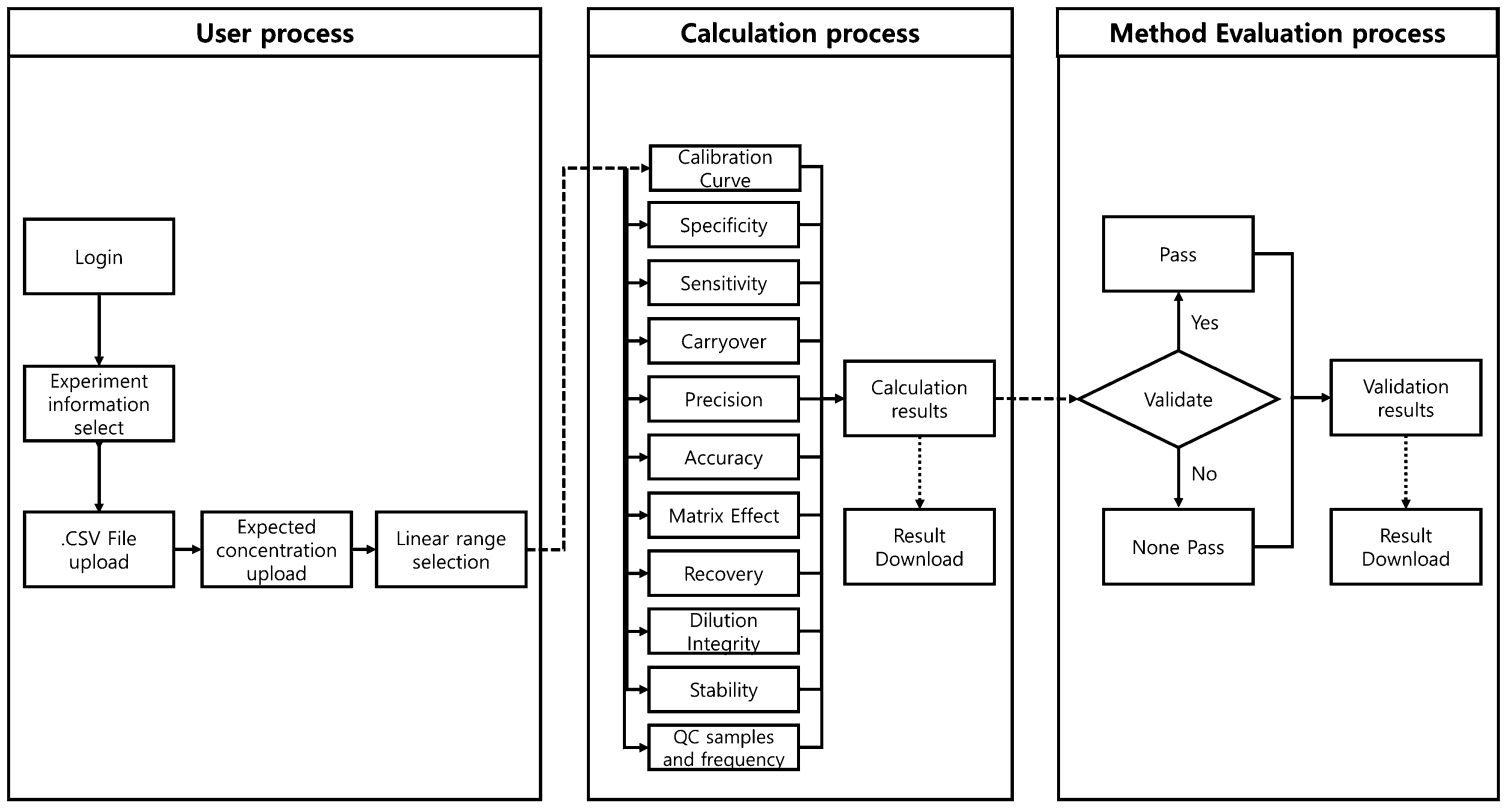
Overview of M-MVP. M-MVP is organized into three categories. The first category (User process) includes login, selecting experiment information, uploading data, filling in expected concentrations, and Linear range selection. In the second category (calculation process), 11 categories are automatically calculated and available for download in table format. Finally, the third category (Method Evaluation process) automatically evaluates whether the results of the calculations pass the criteria provided in the country-specific guidelines.

Results pages are divided into two parts: calculation and method evaluation pages (Fig. 2).

**Figure 3:**
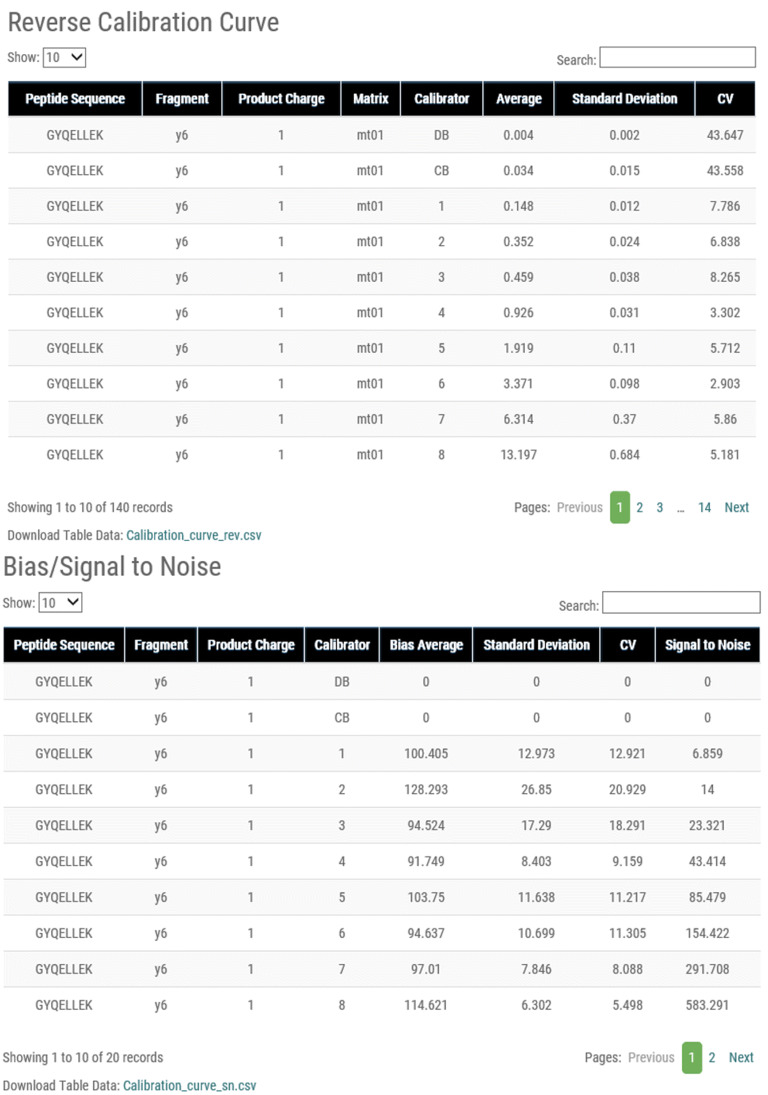
Calibration curve result page. Calculation results of Calibration Curve, Specificity, Sensitivity, Carryover, QC, Precision, Accuracy, Matrix Effect, Recovery, Dilution Integrity, and Stability are shown separately on individual pages in table form. Protein Name, Peptide Sequence, Fragment Ion, Product Charge, Replicate Name, and the results corresponding to the validation criteria are shown.

For calculation pages, the calculated results are shown in table form, in which peptide sequence, fragment, product charge, and replicate name are set as default column descriptions. Calculated values, such as averaged Peak Area Ratio (PAR) and standard deviation, are shown if required by the category (Fig. 3). In the Table, the results are shown in list form in 10 lines and a maximum of 100 rows. To save the results for personal use, M-MVP supports downloading of the table as a csv file.

**Figure 4:**
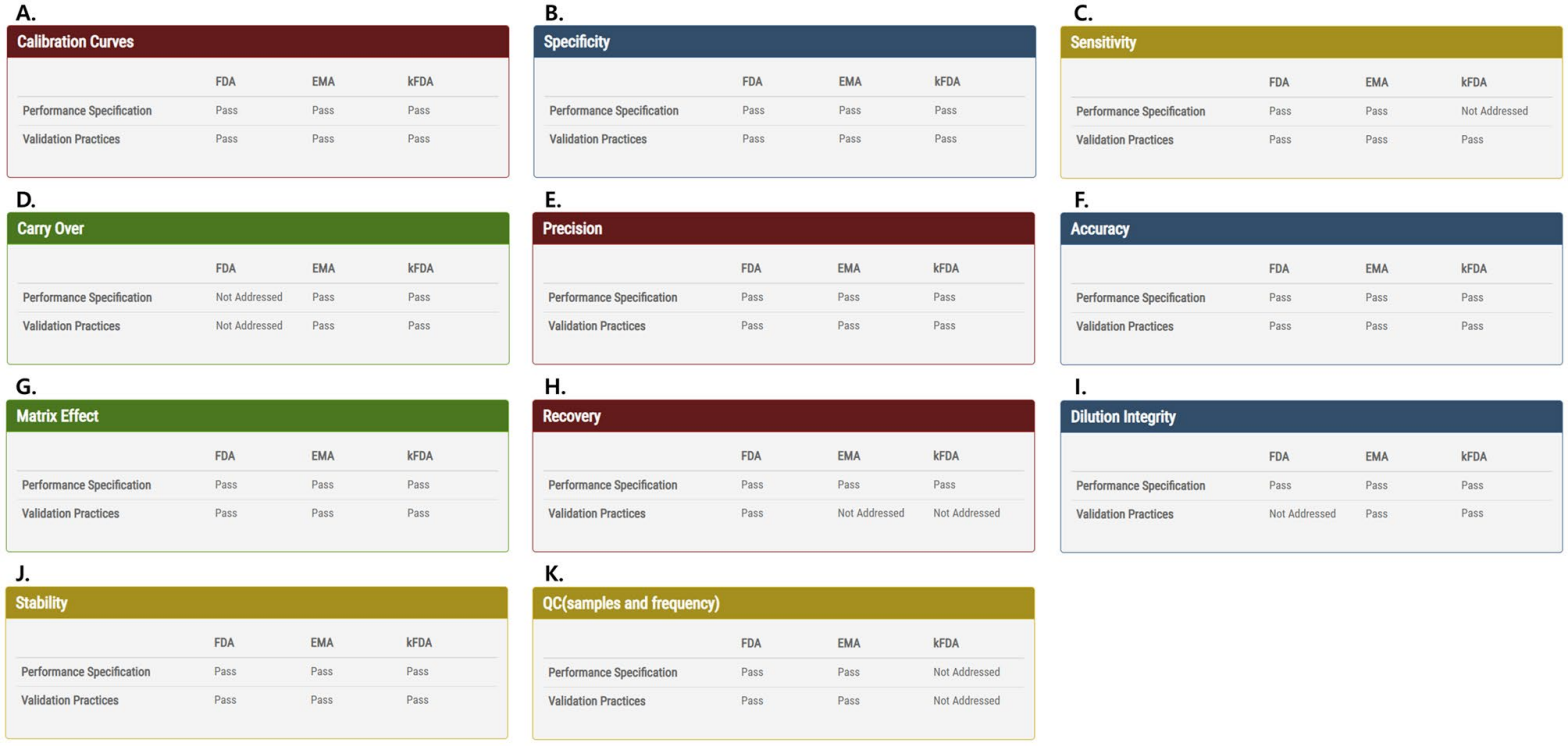
Method evaluation result. Validation results are shown on a single page. For each category, the table will show if it passed or not for the validation standard of the 3 administrations. If none of the administrations has a regulation on a particular category, “not addressed” is shown. (**A**) Calibration Curves, (**B**) Specificity, (**C**) Sensitivity, (**D**) Carry Over, (**E**) Precision, (**F**) Accuracy, (**G**) Matrix Effect, (**H**) Recovery, (**I**) Dilution Integrity, (**J**) Stability, and (**K**) QC validation results are shown as above. The user can reupload his data files for categories that did not pass the standard.

For method evaluation pages, when the user requests the calculation pages, the server will evaluate if the current categories pass the performance specification of three regulatory agencies (FDA, EMA, and KFDA) and will then store the result, along with the uploaded data. The user must examine each category to determine whether M-MVP result for a category satisfies the specified guideline. After examining all 11 categories, the user can check to see if the categories pass the performance specifications and validation practices by displaying “Pass” or “Not Pass” or “Not Addressed” if the regulatory agency did not specify a certain standard (Fig. 4). The evaluation is performed, based on the guidelines from all 3 regulatory agencies, and the results are shown in a single table. When 1 or several categories fail to pass the evaluation, the user must revise the relevant category in the experiment and then upload the revised csv file.

In designing M-MVP on a working server, 1 of the main priorities was to implement an MRM-MS assay for AFP-L3, a well-known diagnostic biomarker of hepatocellular carcinoma^18^. As a working example, the analytical method for M-MVP was verified using the data from the previous assay experiments, because all categories of the method validation experiment had been performed for the assay and already verified with standards from the 3 administrations.

With Skyline output data for the 11 categories, we developed and tested the performance of M-MVP. The performance specifications and validation practices of the categories are embodied in M-MVP and are represented in the resulting tables, in which the format of the input data and guideline details has been adopted from the supplemental tables that were created for the previous study^18^. Further, the performance specifications of the calculations that were performed by M-MVP were compared with those of a manual method using Excel with the same data. The M-MVP calculations agreed with the Excel values up to 4 decimal points, at which point the differences were insignificant. In terms of the time that was required for the performance and validation, M-MVP completed all categories instantaneously, whereas the manual process of calculation and validation in Excel took several hours. All data for the 11 categories of the MRM-MS AFP-L3 assay are in the Tutorial tab, where users can access them for self-education (Supplemental Information).

## Discussion

We developed M-MVP for validating the MRM-MS assay using Skyline data. As an online assay portal, calculations are performed on the server side, which lowers the computational burden on users. Our portal aims to support the analytical validation of the MRM-MS assay. M-MVP is especially effective for processing large sets of MRM-MS data, such as data that are generated with a multi-marker panel assay.

Several issues require further development. For instance, the current build is sensitive to naming parameters and requires that the dataset follow the naming conventions of Skyline-generated .csv files. Therefore, the user must ensure that the uploaded files have the correct naming format that is required by M-MVP. Developing a flexible naming function might decrease the learning curve for users.

Incorporating M-MVP into the external tools of Skyline is another plausible option for development. Implementing the core function of M-MVP into Skyline's external tool would drastically reduce the time that is required for validation of the MRM-MS assay, because the process would run immediately on the Skyline. Even if M-MVP, as an external tool in Skyline, requires more computing power from the user, one could prefer not have to move files from the Skyline to our portal and could perform the entire process on a local computer. This process will ultimately lead to automation of the analytical method validation process of the MRM-MS assay.

Rapid advances in the sensitivity and selectivity of mass spectrometry will result in successful development of MRM-MS-based multi-marker assays. The main purpose of developing M-MVP was to facilitate the introduction of an MRM-MS-based multimarker assay into commercial sectors. Naturally, the assay development process must abide by the multinational guidelines. We hope that M-MVP will accelerate the implementation of the MRM-MS assay in clinical applications by lowering clinical entry barriers.

Furthermore, we expect that M-MVP will be applicable for metabolomics research of small molecules and chemicals, for which relevant assays will require analytical method validation.

## Methods

### Architecture of M-MVP

The SPRING framework, which features a standard Model-View-Controller (MVC) that is ideal for webserver applications, was the fundamental component of the server that formed the infrastructure of M-MVP. As part of the MVC model, the JAVA controller handles requests and mapping, in which JAVASERVER PAGES works as a dynamic web page, with AJAX for asynchronous web applications and file upload for user interfaces**.** The MYBATIS framework was used to handle Structured Query Language (SQL) statements for data calculation and storage to the mySQL Database Server. BOOTSTRAP is a general open source JQUERY library for web page user interface design. BOOTSTRAP was used to design the table format in M-MVP that was used for cases in which evaluation guidelines and output files were presented in table form. GRADLE is a build automation system that automatically manages libraries and builds a Web Application Archive (WAR) file that is deployed on a TOMCAT server. Because floating-point calculation varies by programming language, data calculation and validation were performed exclusively with SQL queries to ensure consistency.

**Figure 5:**
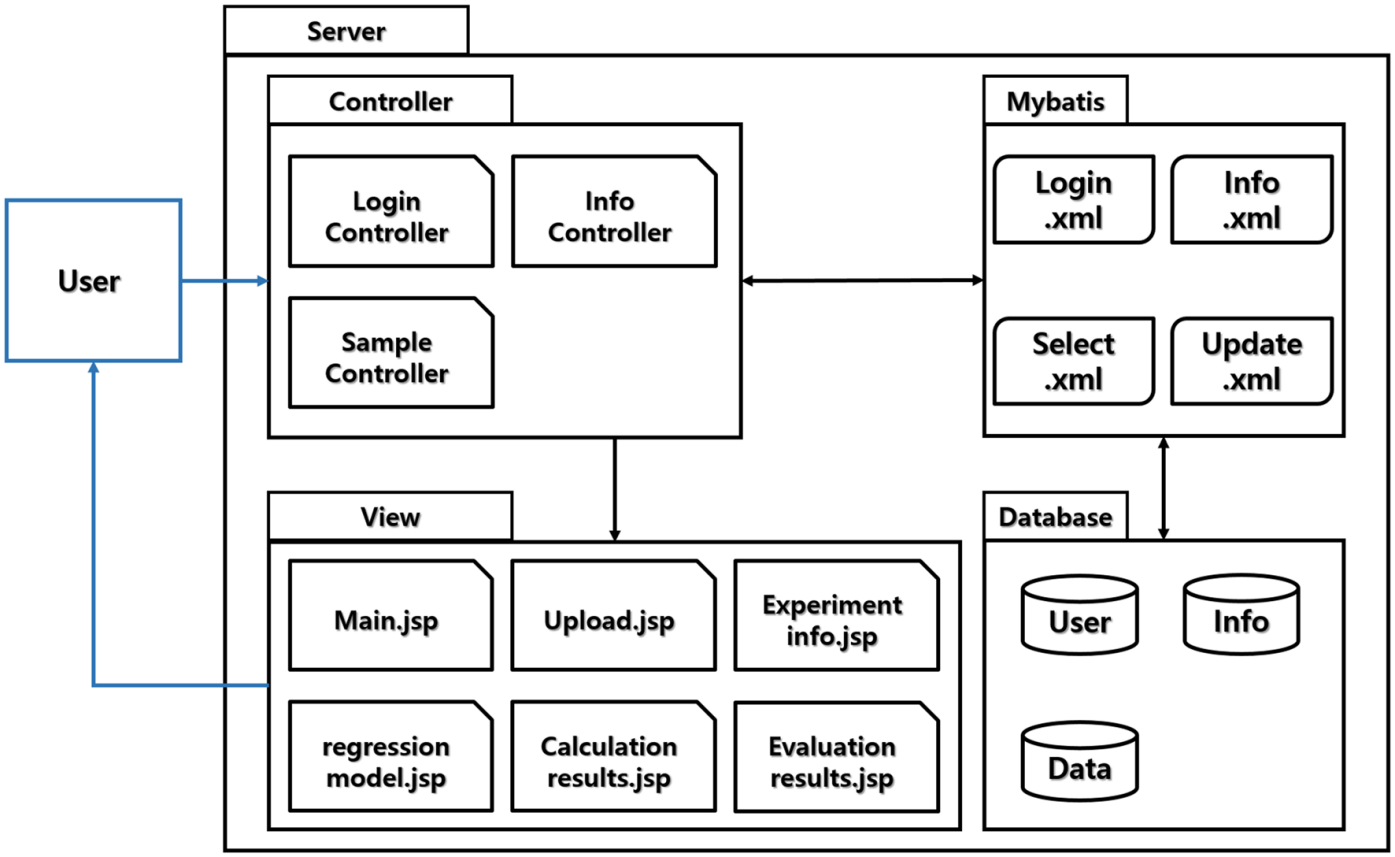
Architecture of M-MVP portal.

To specify the architecture of the portal (Fig. 5), Controllers, which are the main java files that control all input–output commands, are divided into 3 categories. LoginController.java handles login-related requests from the client, whereas InfoController.java handles experimental data information of the logged-in user. All other requests for webpage loads or calculation functions are handled in SampleController.java. Upload and calculation of user data require the server to connect with the database server, which are controlled by the Mybatis package. The xml files package contains SQL queries, which are called by the controller under a user’s request. The database contains individual schema for user information, experimental information, and uploaded data. All webpages are composed with JSP, and the design is composed with html and css. Dynatable, an open-source interactive table that uses jQuery, is implanted for the entire table format of the portal.

### Data format

Depending on the analyte and the type of mass spectrometer, data analysis software may vary. Skyline (MacCoss Lab) is the most commonly used data processing software for MRM-MS. We developed M-MVP to accept Skyline output files in csv format, in which the column and analyte names must be in agreement with our specified format. We defined validation categories, such as calibration curve, specificity, sensitivity, carryover, precision, accuracy, matrix effects, recovery, dilution integrity, stability, and QC (samples and frequency), based on guidelines from the FDA, EMA, and KFDA. For all validation categories, step-by-step instructions for adopting the Skyline output file into the format that M-MVP requires are provided. Only with a specific format can M-MVP accept the uploaded Skyline data for calculations and validation.

To develop the portal, we used the entire validation datasets of our previous study (Kim et al.^18^). The datasets were verified to pass the standards of all 3 administrations.

### Calculation and validation method

For calculations, M-MVP extracts light and heavy area values from the Skyline files. PAR and concentration ratio (from the reverse calibration curve data) are used for the linear regression analysis. The user chooses a linear equation that is used by M-MVP to calculate the concentrations of subsequent categories, such as Sensitivity and Recovery. For some categories, the standard deviation and coefficient of variation (CV) of PAR or concentration are calculated. Percent difference between the initial value and measured value is also calculated when required by the guidelines.

The calibration curve presents reverse and forward calibration curve data. The calibration curve falls within the range of measured concentrations by the instrument in which mass spectrometry can show linear measurements. Specificity is assessed by definite signals of analyte and Internal Standard (IS) in blank samples. Sensitivity is assessed by the first calibrator or lower limit of quantification (LLOQ), when the method provides acceptable precision and accuracy. Evaluation of carryover is assessed by injecting blank samples following a high-concentration sample or samples that are used for upper limit of quantification (ULOQ). Precision and accuracy are assessed by analyzing QC samples. Within-run precision and accuracy are calculated by averaging the concentration of replicates of each target QC concentration on each day, whereas between-run precision and accuracy are calculated by averaging the first run of each target QC concentration across all days. Accuracy values are assessed by dividing the measured concentration by the expected concentration.

For the validation of matrix effects, PAR of a spiked target in each matrix and in neat solution is calculated, and then, the values of all six matrices at the same concentration are averaged. Recovery was assessed as the relative recovery of recovered target to input target in terms of PAR at each QC concentration. Dilution integrity evaluates whether dilution affects the precision and accuracy and is assessed by calculating the change in concentration resulting from sample dilution. For stability validation, M-MVP sets day-0 as the standard point and compares the measured values at day-0 with other conditions at every QC concentration. In terms of QC (samples and frequency), the accuracy of QC samples is assessed in at least 5% of the total number of patient samples. This method assures that sample preparation and storage do not affect sample concentration. All aforementioned calculations are performed by SQL queries in the MySQL server; calculation methods are summarized in Table 1. M-MVP provides an assorted list of validation standards that are issued by the 3 regulatory agencies as references.

**Table 1:** Specific value description and formula for the 11 categories.

No	Category	Values	Explanation
1	Calibration curve	Signal to noise	$\frac{\text{PAR} \;calibrator}{\text{PAR}\;zero\;sample}$
2		Bias	$\frac{\text{Slope} \; \text{of}\;\text{each}\; \text{matrix} -\text{Mean} \; \text{slope}}{\text{Mean}\; \text{slope}} \times 100$
3	Specificity	Interference (%)	$\frac{\text{Peak}\; \text{Area} \; blank \; sample}{\text{Peak} \; \text{Area} \; calibrator \;1} \times 100$
4	Sensitivity	Signal to noise	$\frac{\text{PAR} \;calibrator \;1}{\text{PAR}\; zero \; sample}$
		Precision	CV of calibrator 1
		Accuracy	$\frac{{{\text{Measured}}\;{\text{concentration}}}}{{{\text{Expected}}\;{\text{concentration}}}} \times 100$
5	Carryover	Carryover	$\frac{\text{Peak}\; \text{Area}\;blank\;sample}{\text{Peak}\;\text{Area}\; calibrator\; 1} \times 100$
6	Precision and accuracy	Intra-day CV	Averaging the mean values of the each replicates on each day
		Inter-day CV	Averaging the first replicate of each day
		Recovery	Measured concentration/expected concentration × 100
		Total CV	$\sqrt{{CVinter}^{2}+{CVintra}^{2}}$
7	Matrix effect	Matrix effect	$\frac{\text{Serum}\;\text{matrix}}{\text{Buffer}\;\text{matrix}} \times 100$
8	Recovery	Recovery	$\frac{\text{Matrix spiked into serum at the beginning of assay}}{\text{Matrix} \; \text{spiked during the elution step}} \times 100$
9	Dilution integrity	Accuracy	(Dilution corrected concentration – Neat concentration)/Neat concentration × 100
10	Stability	Recovery	$\frac{\text{Concentration}}{0 \text{-day}\; \text{Concentration}} \times 100$
11	QC (samples and frequency)	Accuracy	$\frac{\text{Measured} \; \text{concentration}}{\text{Expected} \;\text{concentration}} \times 100$

### Implementation

A schematic of the M-MVP pipeline is shown in (Fig. 2). The user is required to log in as a guest or with the registered ID and password that are issued by the administrator. The process for the analytical method validation is accessible from the validation tab on the main page of M-MVP (Fig. 1). On the following page, users are required to input information regarding their experiment and corresponding validated data. Once the user provides all required fields, a unique ID of the experiment is generated and is applied by the user to access and proceed with uploading the data. We designed the portal with a login function for two critical obstacles when evaluating the method: to avoid uploading the same files each session if the user did not pass the designated criteria in the first attempt and to manage each distinct experiment easily under the designated login ID. We also developed a guest login feature that does not require a login but is a nearly identical login process that differs by requiring users to remember their experiment ID. Uploaded data are stored and deleted after 1 week from the initial upload.

**Figure 6:**
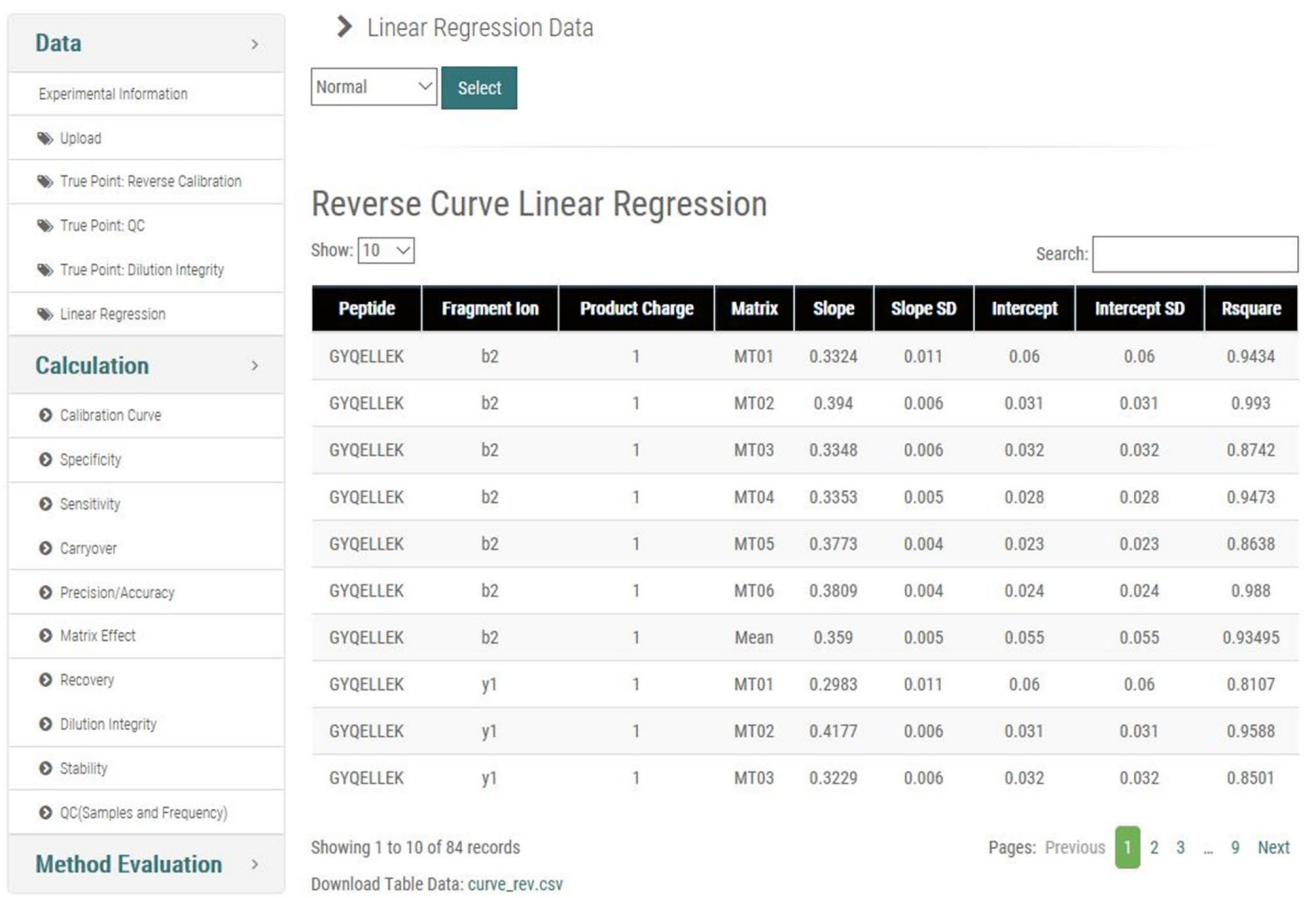
Linear regression result page. The linear regression equation is the key function of the method validation process. The linear equation that is formed by reverse calibration curve data is used for concentration calculations in other categories. Depending on the circumstance, users must decide to whether to proceed with their calculations in normal or log scale. On this page, the portal provides slope, intercept, and R square values of each equation so that the user can decide which equation is optimal for calculation. The scale that is ultimately selected is used for concentration calculations. Other accessible pages are shown on the left side of the page.

To proceed to the validation step, users operate three separate tasks. Users must upload all experimental data that are relevant to each of the 11 categories; two entries for calibration curve are available for upload. Only the categories with uploaded files will be validated according to integrative multinational guidelines. Although multiple data files for each category can be uploaded, only the most recently uploaded files are used for calculations and validation. This design allows users to re-do one specific category, as opposed to repeating the entire experiment. The next step is to upload the expected concentrations. Accuracy is defined as the closeness of agreement between an assay result (experimental measurement) and the expected concentration (true value). With respect to accuracy measurements, users must input true values for calibration curve, quality control (QC), and dilution integrity in the corresponding web page of M-MVP so that calculations and validation are performed. The last step is for the user to choose the scale of the calibration curve, which is subsequently reflected in the corresponding calculations of the other categories. Under the Data menu, the “Linear Regression” page contains the linearity result for each transition (Fig. 6). The user is given an option to choose a normal, log_2_, or log_10_ scale for calculating the concentration. Once the user chooses a scale, the result is shown instantly in table form. By showing the intercepts, slope, and R^2^ in a table, M-MVP provides flexibility to the user for determining which one should be used to calculate the linear regression. The linear equation that the user selects from the Reverse Calibration Curve page is used for calculating the concentration of other categories. With all steps accomplished, M-MVP processes the calculation and method evaluation and shows the results to the user.

## Supplementary information

Supplementary file1 (DOCX 3213 kb)

Supplementary file2 (ZIP 100 kb)

## Supplementary information

nis available for this paper at 10.1038/s41598-020-67731-x.
