# Supplementary material for: Web portal for analytical validation of MRM-MS assay abided with integrative multinational guidelines
Source: Sci Rep. 2020 Jul 2;10:10848. doi: 10.1038/s41598-020-67731-x (PMC7331696; doi:10.1038/s41598-020-67731-x)
Supplement: Supplementary file 1 — Supplementary file1 (DOCX 3213 kb) [file 41598_2020_67731_MOESM1_ESM.docx]

**Web portal for analytical validation of MRM-MS assay abided with integrative multinational guidelines**

Jaenyeon Kim^1,2,#^, Injoon Yeo^1,2,#^, Hyunsoo Kim^2,#^, Areum Sohn^2^, Yoseop Kim^1^, Youngsoo Kim^1,2,3,4,*^

^1^Interdisciplinary Program of Bioengineering, Seoul National University College of Engineering, Seoul, Korea; ^2^Institute of Medical and Biological Engineering, MRC, Seoul National University, Seoul, Korea; Departments of ^3^Biomedical Sciences and ^4^Biomedical Engineering, Seoul National University College of Medicine, Seoul, Korea

^#^ **These authors contributed equally to this work.**

*** Corresponding authors (Contact Information):**

Youngsoo Kim, PhD

Department of Biomedical Engineering, Seoul National University College of Medicine

103 Daehang-ro, Jongno-gu, Seoul 03080 Korea

Tel: 82-2-740-8073, Fax: 82-2-741-0253, E-mail address: [biolab@snu.ac.kr](mailto:biolab@snu.ac.kr)

**Keywords:** analytical method validation, multiple reaction monitoring-mass spectrometry, biomarker assay, web portal, proteomics, biomarker

**DataSET Description**

The analytical method validation experiments aimed to meet the requirements (validation practices) of 3 sets of guidelines [US Food and Drug Administration (FDA), European Medicines Agency (EMA), and Korea FDA (KFDA). These items covered calibration curve, analytical specificity, analytical sensitivity, carryover, precision and accuracy, matrix effect, recovery of immunoprecipitation, dilution integrity, stability, and quality control of the samples and frequency.

The descriptions for the following methods are abstracted and summarized from the supplemental methods in Supplemental DATA in Kim, H. *et al* (2018) [1].

**Calibration curve**

The reverse calibration curve was used to calculate the concentrations of the samples, which contained stable isotope-unlabeled internal standard protein analog. Conversely, the forward calibration curve was used to calculate the concentrations of patient samples that contained stable isotope-labeled internal standard protein analog.

The limit of detection (LOD) and limit of quantification (LOQ) were determined, based on the mean values from the zero samples plus 3 and 10 times the standard deviation (SD), respectively. To determine the LLOQ, samples at 5 concentrations (0.005–0.500 ng/mL) that were close to the predetermined LOD were analyzed in 3 replicates in 1 d. The lowest concentration that met the precision [coefficient of variation (CV) < 20%], accuracy (within ± 15% of target), and signal-to-noise ratio (S/N > 5) criteria was selected as the LLOQ. The upper limit of quantification (ULOQ) was defined as the highest concentration in the reverse calibration curve that met the following criteria: precision CV < 20% and accuracy within ± 15% of target.

**Data_forward_calibration_curves.csv and Data_reverse_calibration_curves.csv were used to perform “Calibration curve.”**

**ANALYTICAL Specificity**

Analytical specificity was examined by comparing the concentrations of endogenous analyte and internal standard in the blank samples with the LLOQ (calibrator 1). To elaborate, interference of the analyte was calculated as the percentage of the peak area of labeled peptide in the blank samples with respect to the peak area of labeled peptide in the samples at the LLOQ. For the internal standard, interference was calculated as the percentage of the peak area of unlabeled peptide in the blank samples with respect to the peak area of the unlabeled peptide in the samples at the LLOQ.

**Data_specificity.csv was used to perform “ANALYTICAL Specificity.”**

**ANALYTICAL Sensitivity**

The LLOQ was defined as the lowest concentration on the reverse calibration curve (calibrator 1) that could be measured with a range of precision of < 20% and an accuracy of 80% to 120% of the target concentrations with an S/N ratio of at least 5. For calculation of the S/N ratio, the signal and noise were represented by the peak area ratios of calibrator 1 and the zero sample (non-blank sample) in each matrix.

**Data_sensitivity.csv was used to perform “ANALYTICAL Sensitivity.”**

**Carryover**

Carryover was evaluated similarly as analytical specificity, except that the blank samples were analyzed sequentially after the sample at the ULOQ (calibrator 8).

**Data_carryover.csv was used to perform “Carryover.”**

**Precision and Accuracy**

Precision and accuracy were calculated by analyzing 4 quality control (QC) samples. Four QC samples were prepared by spiking stable isotope-labeled protein analog into the matrix at the following concentrations: QC 1 (LLOQ, 0.051 ng/mL), QC 2 (3 × LLOQ, 0.154 ng/mL), QC 3 [(LLOQ + ULOQ) /2, 2000 ng/mL], and QC 4 (0.9 × ULOQ, 3600 ng/mL). Each QC sample was analyzed in 6 replicates per day for 6 d. The CV was used to report the intraassay and interassay precision. The intraassay and interassay recovery relative to the target should also be within ± 15% for all concentrations except for QC 1 (± 20%).

The CV values and concentrations of each of the 6 replicates on a given day were calculated and averaged. Subsequently, these mean CV values and concentrations for each day were averaged to calculate the overall precision and intraassay recovery. The first replicate of each day was averaged to calculate the precision and inter-assay recovery.

**Data_precision and accuracy.csv was used to perform “Precision and Accuracy.”**

**Matrix effect**

First, the matrix effect was evaluated by preparing 8 calibrators and 4 QC samples from each of the 6 matrices. In contrast, the QC samples were analyzed in 6 replicates for a given QC sample in a particular matrix. The matrix effects were calculated by comparing the peak area ratio (labeled to unlabeled) of the analyte in the serum matrix with that in neat solution at the same concentrations. The resulting ratio was expressed as %matrix effect.

Second, the recovery of spiking was calculated for the 4 QC samples, based on the concentrations in the zero samples. Recovery of spiking is used to determine whether a systemic shift occurs in the analytical signal of an analyte due to a matrix effect.

Third, the matrix effect was evaluated in a matrix mixing experiment. If significant sample-specific matrix effects were present, the measured concentrations will not agree with the expected concentrations. To confirm this, 2 serum samples were mixed at various proportions (100:0, 80:20, 60:40, 40:60, 20:80, and 0:100) to evaluate the linearity of the patient samples.

Fourth, endogenous compounds in blood, such as hemolysis, lipemia, and icterus (problem samples), can elicit a matrix effect that suppresses or enhances ionization. To assess the extent to which such abnormalities affected the quantitative results of patient samples, target concentrations were measured in lipemic, hemolyzed, and icteric samples, mixed at a 1:1 ratio with “normal” serum sample.

Lastly, exogenous compounds were evaluated using 2 types of tubes [serum separation tubes (SSTs, serum) and tri-potassium-ethylenediaminetetraacetic acid (K3-EDTA, plasma)] to store blood from 6 patients with HCC.

**Data_matrix effect.csv was used to perform “Matrix effect.”**

**Recovery OF IMMUNOPRECIPITATION**

The recovery after enrichment by immunoprecipitation was determined in 4 QC samples of 6 serum matrices. Stable isotope-unlabeled and labeled protein analogs were added before the AFP enrichment step in 1 set of samples, whereas it was added to another set after the enrichment step. Recovery of immunoprecipitation was expressed as the relative recovery in peak area ratio of the enriched versus unenriched samples.

**Data_recovery.csv was used to perform “Recovery OF IMMUNOPRECIPITATION.”**

**Dilution integrity**

The effect of sample dilution on precision and accuracy was assessed by spiking the blank sample with stable isotope-labeled protein analog at 8000 ng/mL (above the ULOQ) and measuring the resulting concentrations at various dilutions, established by diluting a blank sample by 5 factors (5, 150, 500, 2500, and 20,000) that were within the dynamic range of the assay. The dilution-corrected concentrations were compared with the initial concentration (8000 ng/mL).

**Data_dilution integrity.csv was used to perform “Dilution integrity.”**

**Stability**

The stability in storage was evaluated to determine the acceptable storage conditions. Stability studies were conducted with 4 QC samples under various storage conditions. To assess short-term storage stability, the samples were stored on the benchtop at room temperature and in an autosampler at 4°C for up to 7 d. Long-term stability was examined by storing the samples in a freezer at -20°C and -70°C for up to 28 d. Freeze-thaw stability was also determined in the 4 QC samples. Sample stability was calculated as the mean difference from the baseline value (0 d or 0 cycles).

**Data_stability.csv was used to perform “Stability.”**

**Quality control of samples and frequency**

Four QC samples were analyzed after every 20 individual samples. A total of 400 individual samples were analyzed, with 20 sets of QC runs (5% of total patient size for each QC concentration), meeting the guideline requirements that QC runs constitute at least 5% of the total patient size.

**Data_quality control.csv was used to perform “Quality control of samples and frequency.”**

**Referenses**

[1] Kim, H., *et al.* Clinical Assay for AFP-L3 by Using Multiple Reaction Monitoring-Mass Spectrometry for Diagnosing Hepatocellular Carcinoma, *Clinical Chemistry 2018*; 64(8): 1230-1238

**M-MVP user guideline**

The following tutorial is designed to guide the user through the entire process of the analytical method validation, step by step.

The M-MVP assay portal is designed to ease the burden of analytically validating MRM-MS assays. Users can validate experimental data by uploading Skyline data pertaining to the 11 categories specified by the FDA, EMA, and kFDA. This tutorial will cover **(1) Skyline Data Process, (2) Sign up and Login Process, (3) Experimental Information, (4) File Upload, (5) Expected Concentration Upload, (6) Linear Equation Selection, (7) Calculation Result Pages, (8) Evaluation Result Page, and (9) Re-Validation Process.**

**Step 1: Skyline Data Process (Skyline data format settings)**

**▪ Replicate Name editing**

Replicate Names must follow the specified format shown below:

• In the **Edit menu**, click on **Manage Results.**

• Click **Rename** to change replicate names.


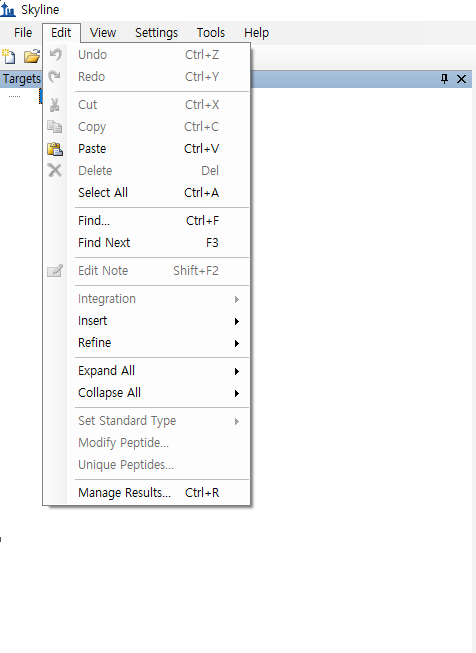

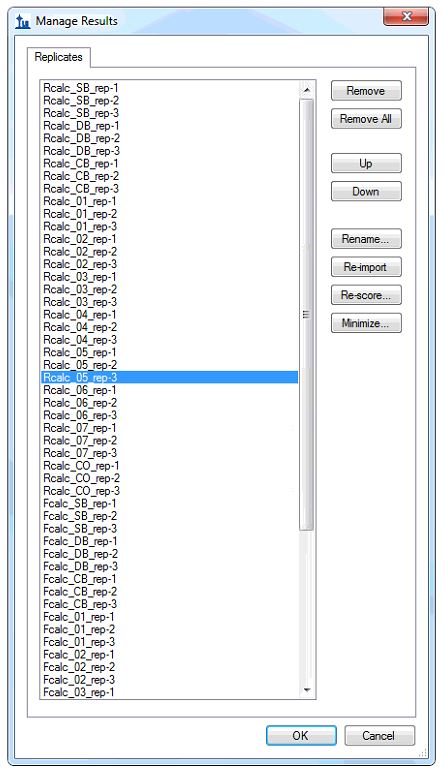


Supplementary Figure S 1

Replicate Names are crucial for proper function of the assay portal. The user must provide the appropriate input for the condition of a sample, which may vary from category to category. Information that is added in Replicate Names is separated by “_” so that the portal can extract text. For **Calibration Curve, Specificity, Sensitivity, Carryover, QC, Matrix Effect,** and **Recovery**, a Replicate Name must contain Matrix Number and Calibrator Number. For example, “MT01_01” indicates the first calibrator of matrix 1. For **Precision** and **Accuracy**, prior to matrix and calibrator information, the user must add information pertaining to the day on which the experiment was performed in a straightforward and intuitive manner—for instance, “1-days_QC01”. **Dilution Integrity** contains the dilution factor “DF_5,” which indicates a dilution factor of 5. For **Stability**, Replicate Name contains temperature, day of experiment, and calibrator, such as “RT_01-day_QC1.”

**▪ Creating a Custom Report**

After creating the custom report template for the Skyline file, perform the following steps:

• In **File menu**, choose **Export** and click **Report.**

Skyline will present the Edit Report form that already contains the fields from the "**Transition Results**” report template, as shown below:

• Choose **Transition Results**, and click **Edit list** in Export Report.

• Choose **Transition Results**, and click **Edit** in Manage Views.

**
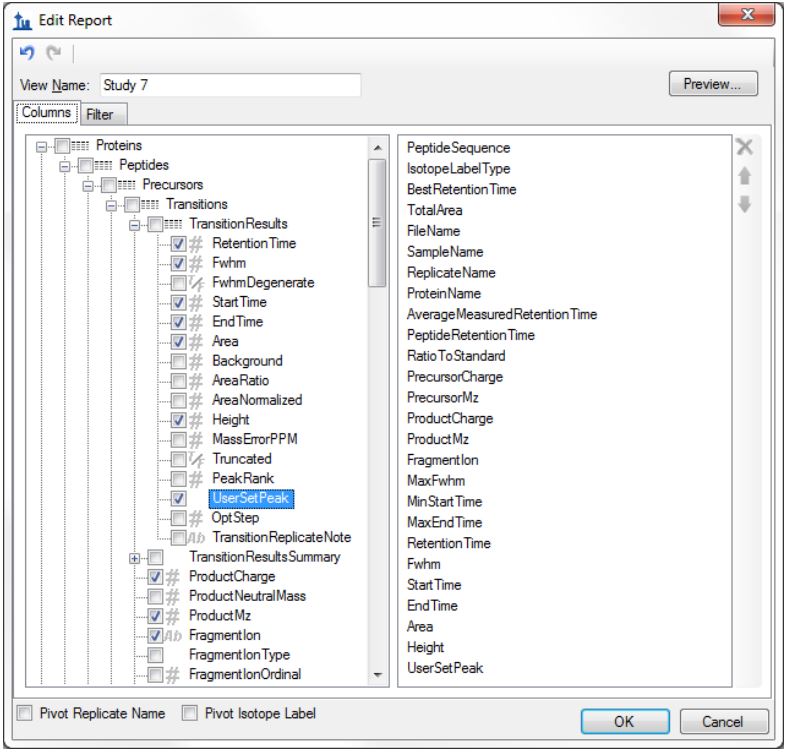
**

Supplementary Figure S 2

• Check the sequence of the following 11 columns: **Protein Name, Peptide Sequence, Replicate Name, Replicate Name, Precursor Charge, Product Charge, Fragment Ion, Precursor Mz, Product Mz, Retention Time,** and **Area.** These columns and their order must be the same as shown above. The column order can be changed using Delete, Moving up, and Moving down on the right side of the Edit Report window.

• Check on **Pivot Isotope Label.**

• On the **File menu**, choose **Export,** click **Report,** choose **Transition Results**, and click **Export.**

When reports are successfully created, the resultant csv file should have the format shown below:


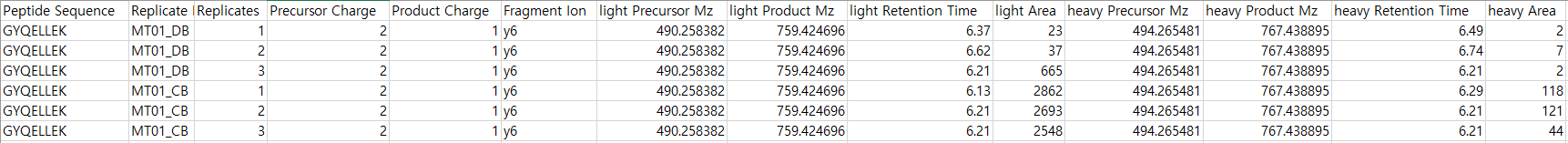


Supplementary Figure S 3

Column format and replicate name need to be in the correct order; otherwise, the portal will not recognize the data for subsequent calculations and validation.

**Step 2: User identification Process**

For this part of the tutorial, our AFP-L3 data are used for demonstration purposes. Sample data are available for download from the **Tutorial** tab.


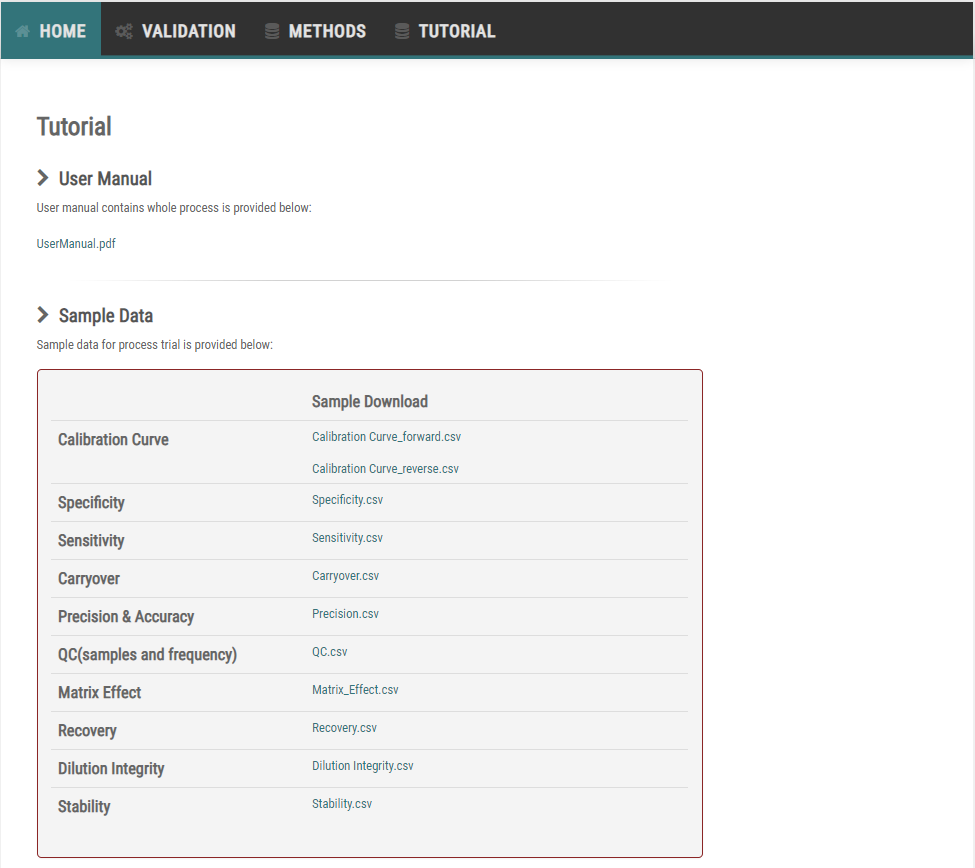


Supplementary Figure S 4

This tutorial will use results from the AFP-L3 assay experiment previously performed as an example. The example files can be retrieved from the download page. Users will acquire a stronger understanding of this tutorial by using the sample data. The amino acid sequence of alpha-fetoprotein (AFP) target peptides is GYQELLEK (indicative of total AFP level) and VDFTEIQK (indicative of AFP-L3 level), which are used to quantify the target protein. As possible target for the target VDFTEIQK peptide, we enlisted b2, y1, y2, y5 fragment with 1+ charge, y6 fragment with 1+ and 2+ charges for the GYQELLEK peptide, b2, y4, y5, y7 fragment with 1+ charge, and y6 fragment with 1+ and 2+ charges. Finally, we chose the y6 fragment ion for quantifying the two peptides (GYQELLEK and VDFTEIQK peptides). Experiments based on the 11 categories of the FDA, EMA, and KFDA guidelines were performed, and the data are provided in this tutorial for users to conduct practice runs.

In the following sections, the tutorial will cover the guest and login processes.

2-1 Guest Process

To process as a guest, users only have to:

• Move the cursor to **Validation**, then **Data,** and click **Guest.**


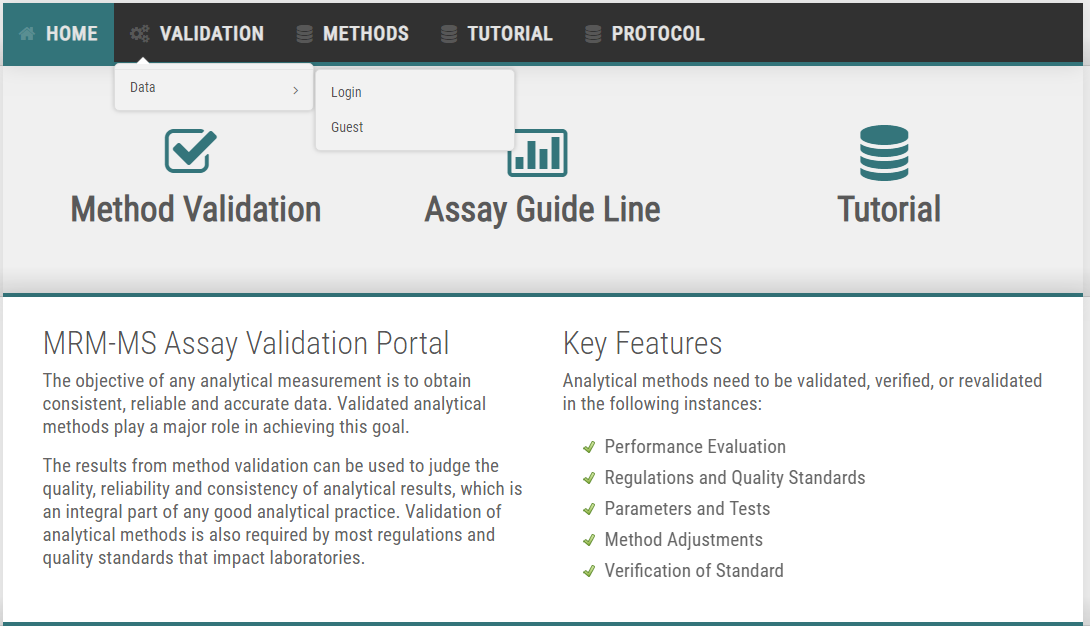


Supplementary Figure S 5

2-2 Sign-up and login process

The login button is located at the top right of M-MVP. Initial sign-up is available on the login page. After signing up, confirmation from the administrator is required for further usage of M-MVP.


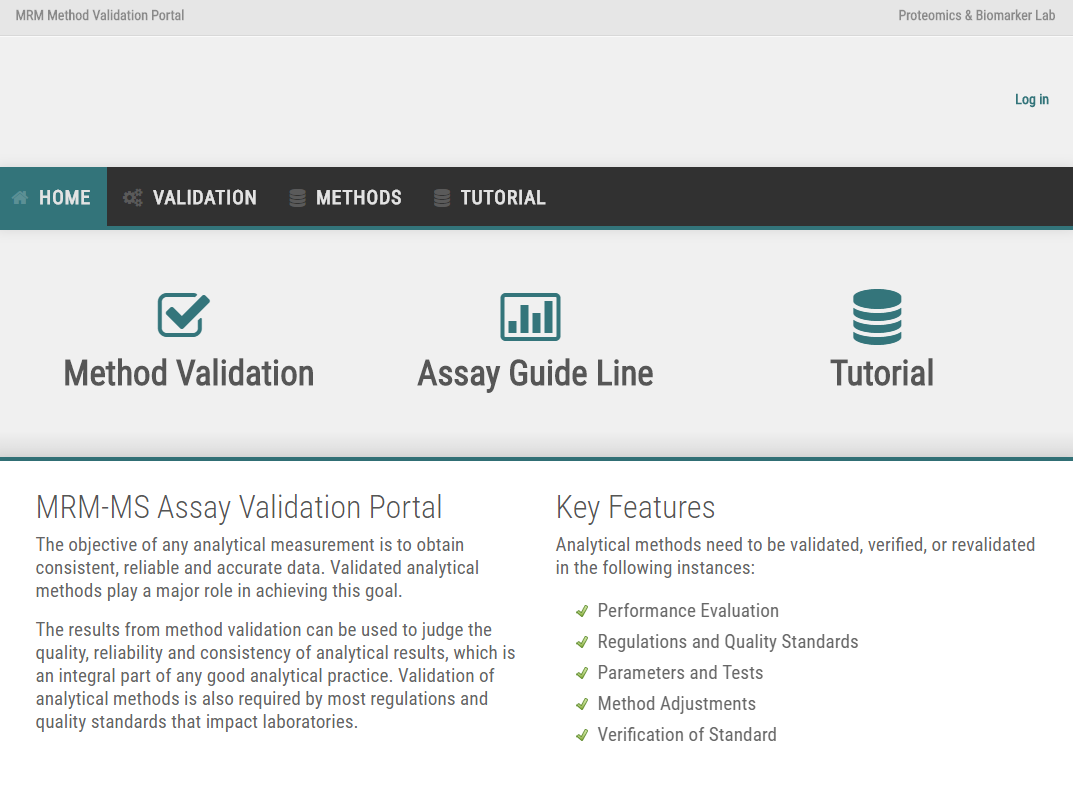


Supplementary Figure S 6

When clicked, the login page will appear as shown below:


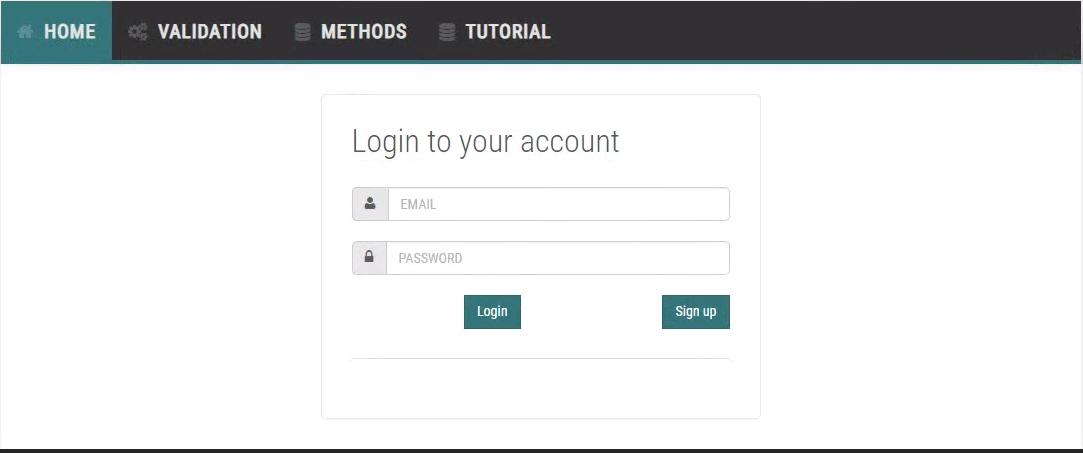


Supplementary Figure S 7

First time users are required to register by clicking **Sign up**.


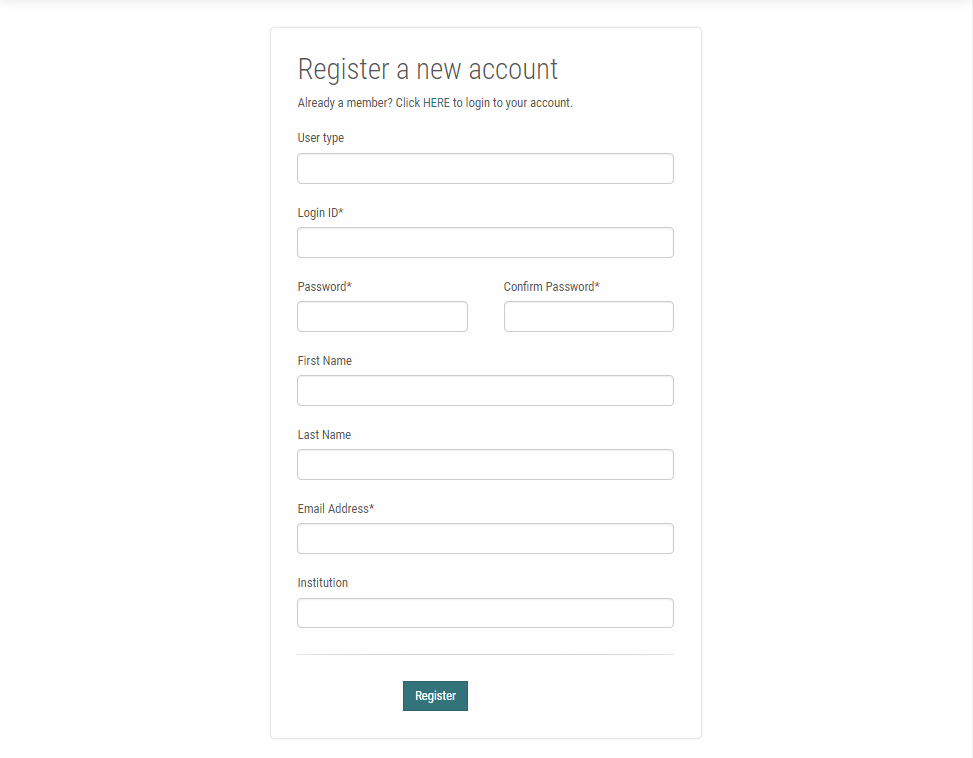


Supplementary Figure S 8

Fill in all required categories and click **Register**. The user will have to wait for administrator approval to use the portal. An email notification will be sent to the registered user indicating that the portal is accessible. Once approved, the user goes back to the **Login Page**. The user enters the ID and password and clicks the **Login** button. The user will be guided to the main page, with a **Logout** button at the upper right, as shown below.


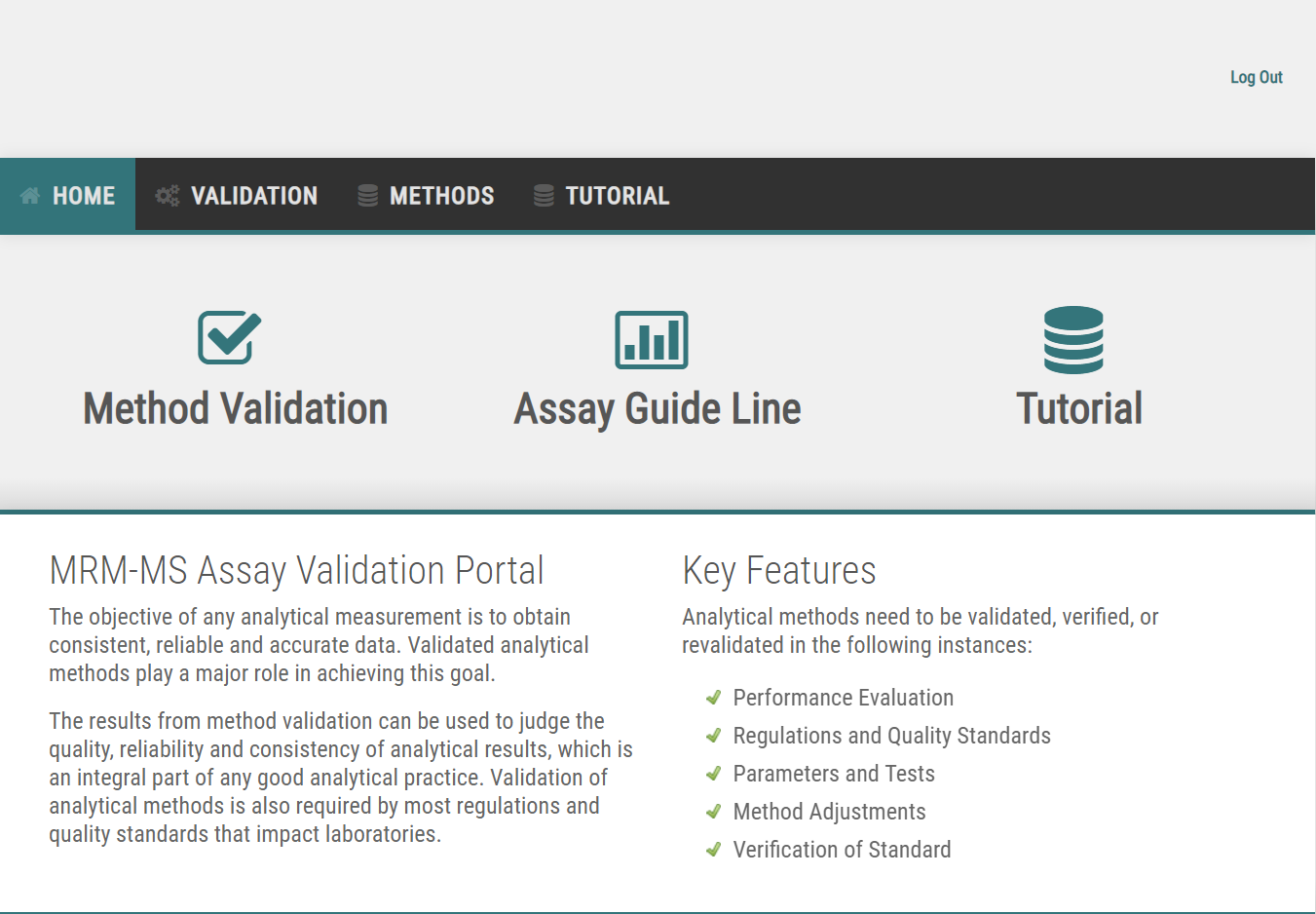


Supplementary Figure S 9

To start method validation, user now have to:

• Move the mouse cursor to **Validation**, then **Data,** and click **Login**.

**Step 3: Experimental Information**

To start M-MVP, first-time users have to register experimental information following our format, as shown in the following steps:


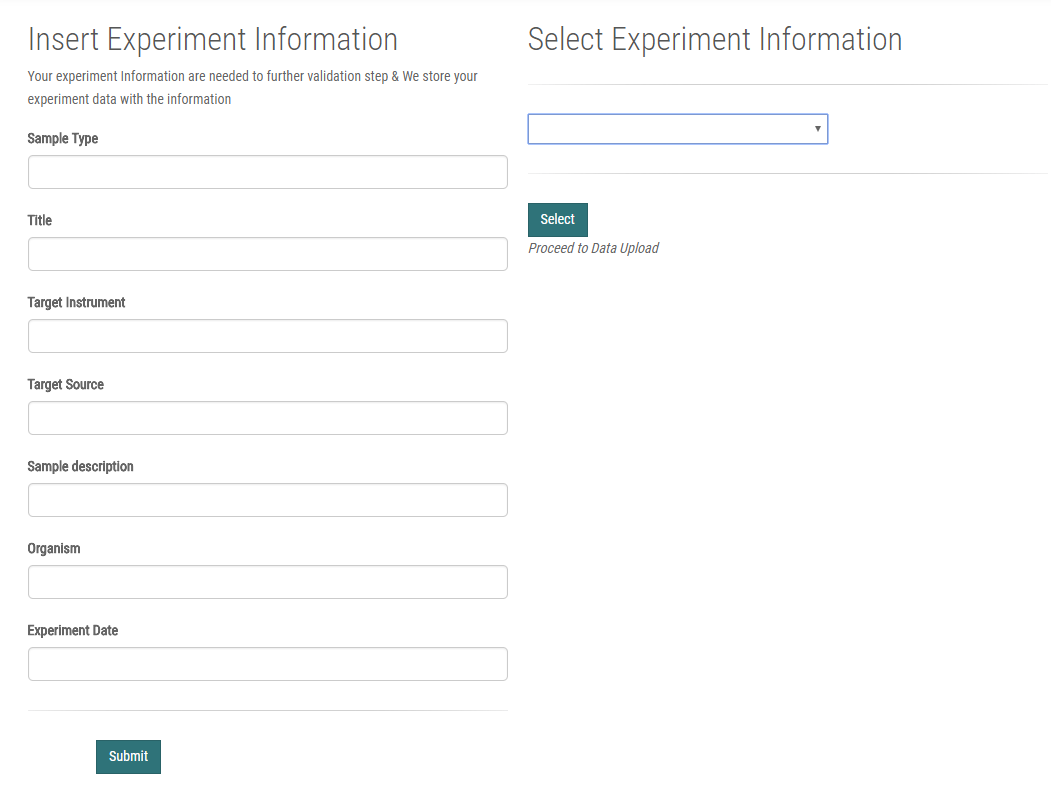


Supplementary Figure S 10

For each experiment, the user will provide experimental information. This step is necessary to distinguish multiple experiments from each other. When submitted, a unique ID is generated at the user’s discretion. Proceed as follows:

• In **Insert Experiment Information**, provide **Sample Type, Title, Target Instrument, Target Source, Sample Description, Organism,** and **Experiment Data.**

• Click **Submit.**

When logged in as a guest, the user will receive a unique experiment ID as a notification message. The portal will now store the experiment ID for the user; thus, the user must remember the experiment ID to proceed to the next step.

The user must input the exact experiment ID manually and click select to proceed. The UI for guests is shown in the figure below.


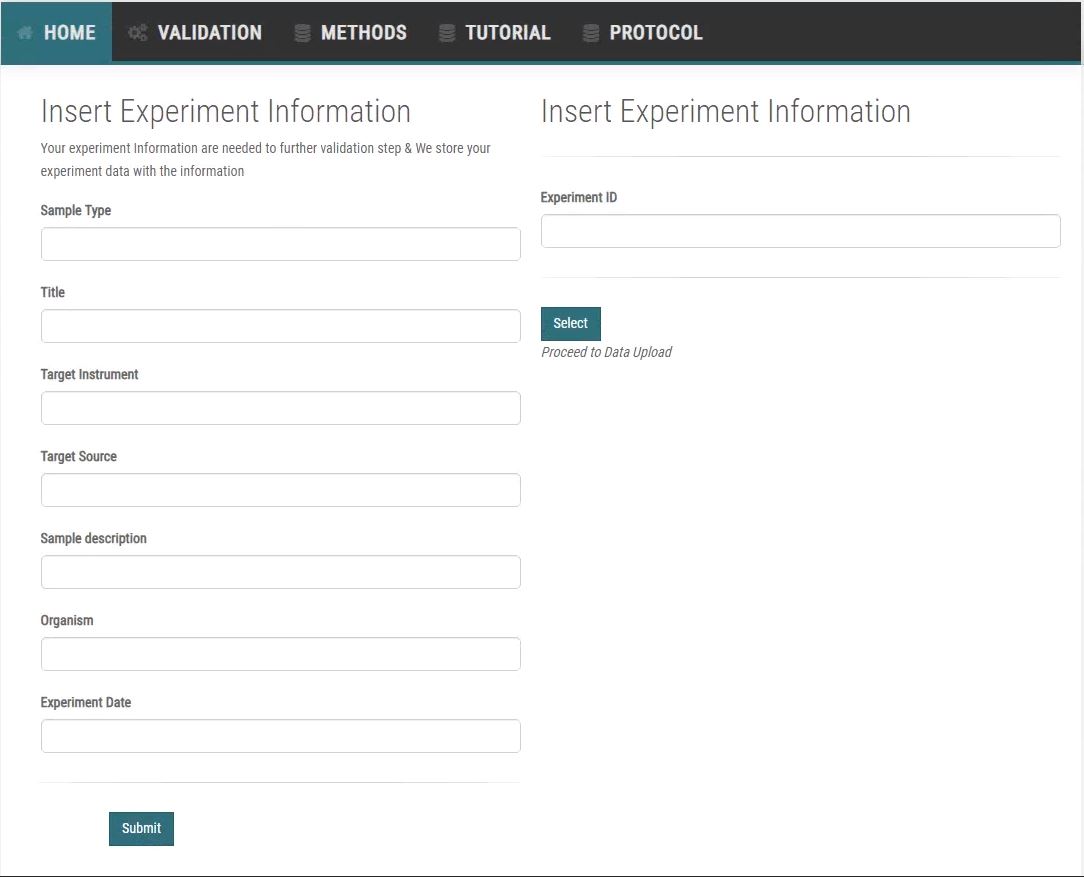


Supplementary Figure S 11

For users who have signed up and logged in:

• Every time a new form is submitted, a new unique ID will be shown in an option box in **Select Experiment Information**.

• Choose the ID of the experiment to be performed.

• Click **Select.**

For example, from the following options in **Select Experiment Information**, there are four experiment IDs that the user has registered.


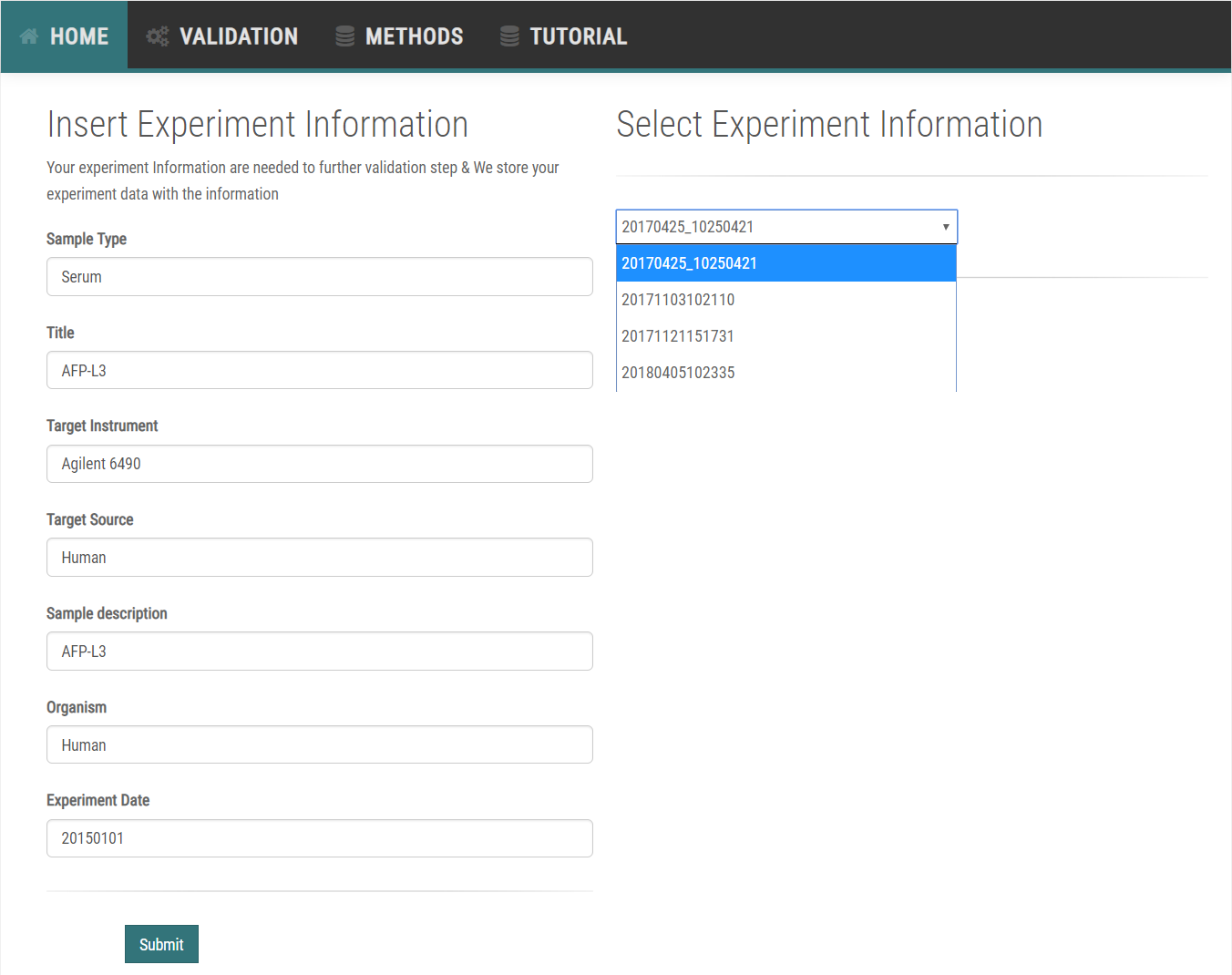


Supplementary Figure S 12

Input all required fields and submit. The website will refresh, and the user will be able to see a new ID at the bottom of the ID list:


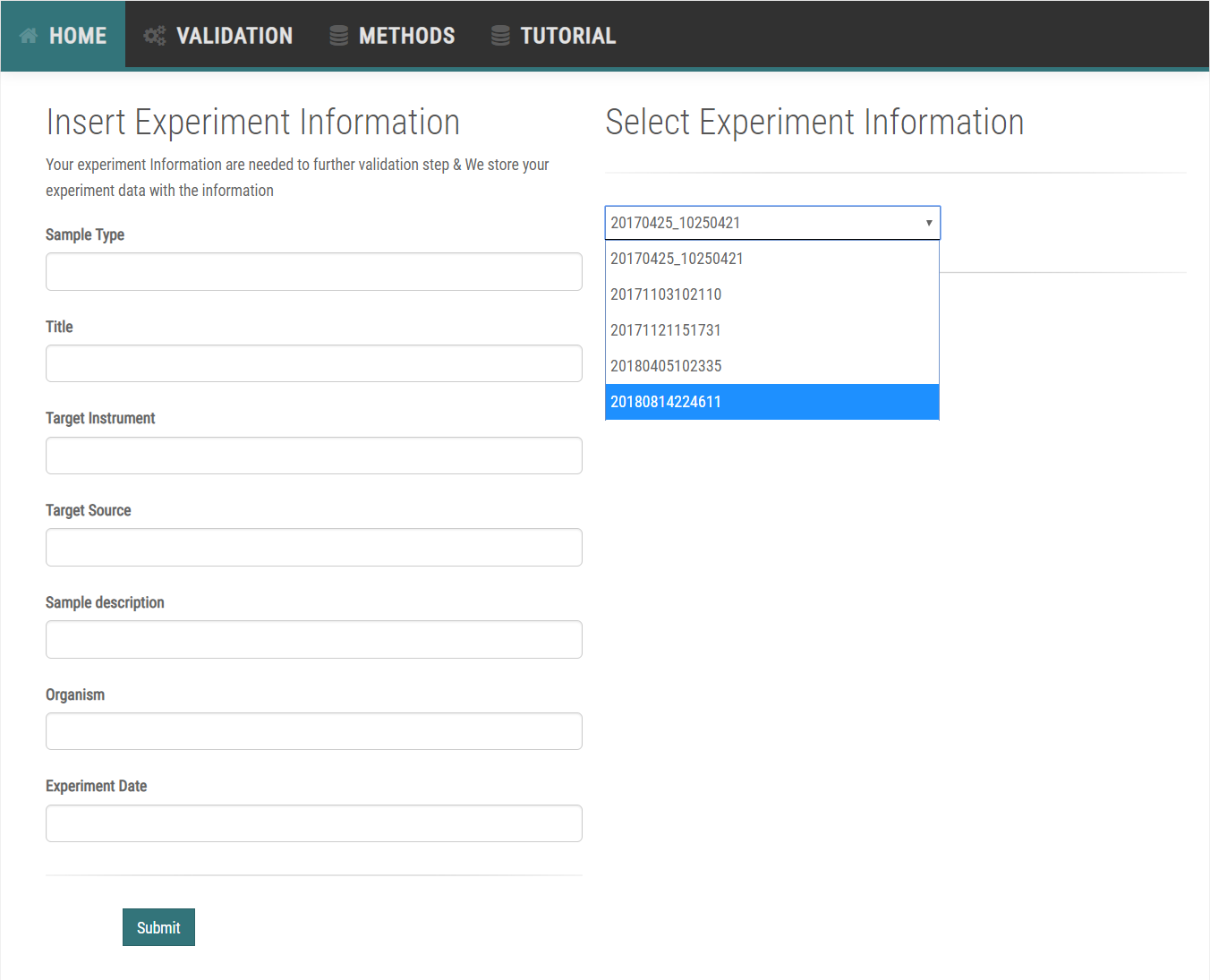


Supplementary Figure S 13

The user selects the experiment to validate by clicking the **Select** button. The most recently submitted experiment ID will appear at the bottom of the list.

**Step 4: File Upload**

On the file upload page, validation process categories are listed, and files can be uploaded separately or all at once. When uploading a new file, previously uploaded data will be discarded, and the new data will be used for calculations and validation.

• Click **Select File** adjacent to the category to which the user wishes to upload a Skyline .csv file.

• Choose the file and click OK.

• After all categories have been filled, Click **FileUpload.**


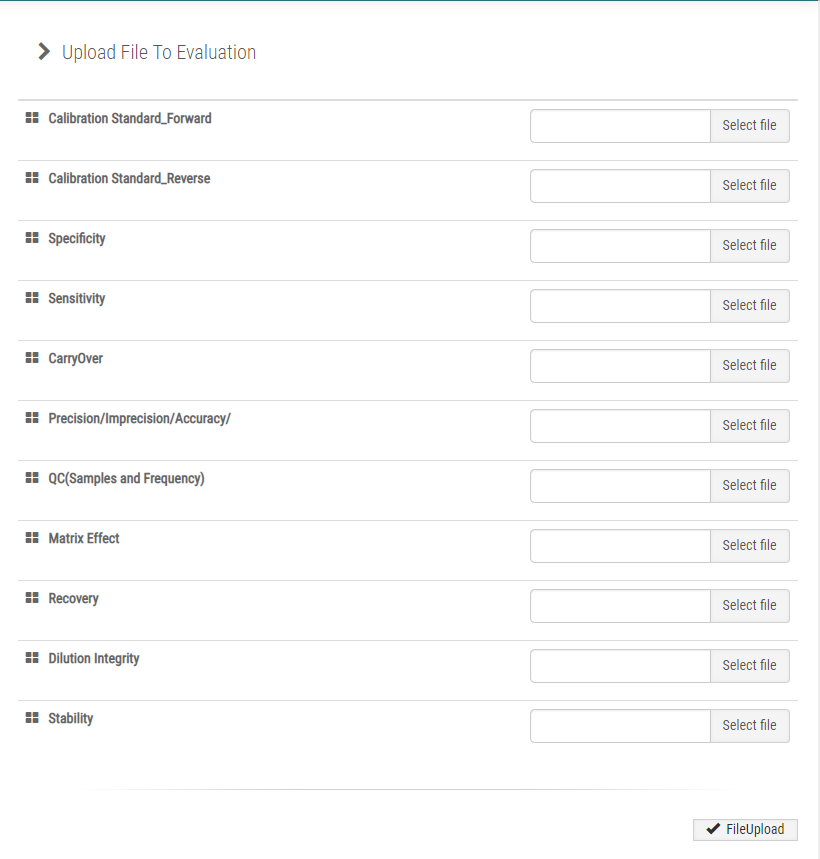


Supplementary Figure S 14

As an example, the user can upload sample data provided in the **Tutorial** tab to the corresponding box as follows:

When selected, the file name will appear in the box. Files can be replaced with the **Change** button or deselected with the **Remove** button. Users can upload multiple files simultaneously. After selecting files, click **Submit**. If there are no issues with the uploaded file, the webpage will refresh, indicating that the upload was successful. The web page will crash if any of the uploaded files is not in the required format.


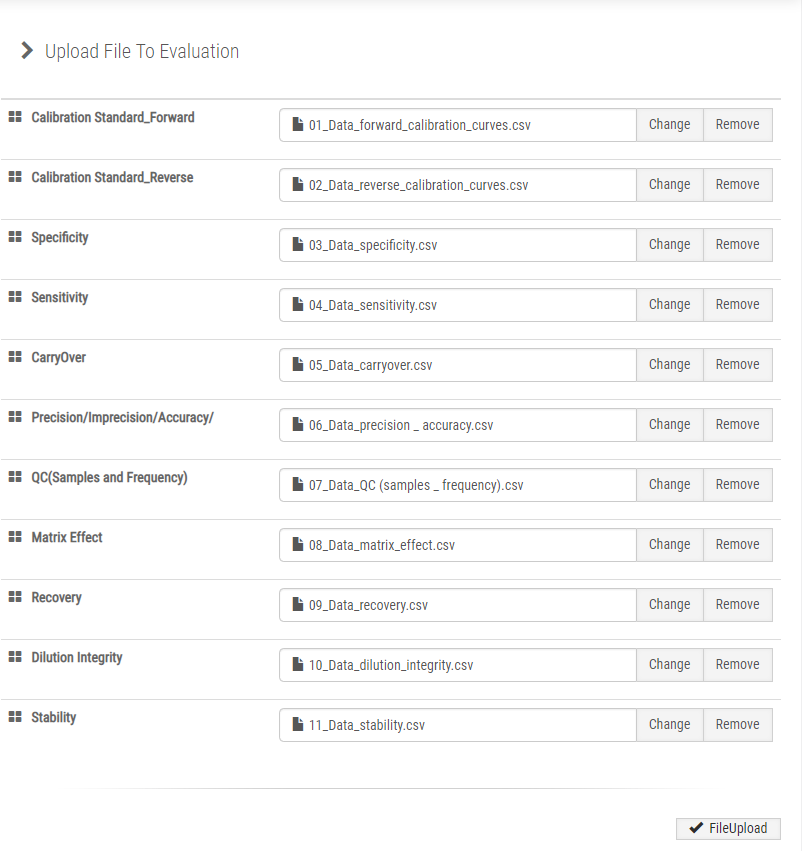


Supplementary Figure S 15

The page will redirect to a blank upload page as shown below if the files were successfully uploaded**.**


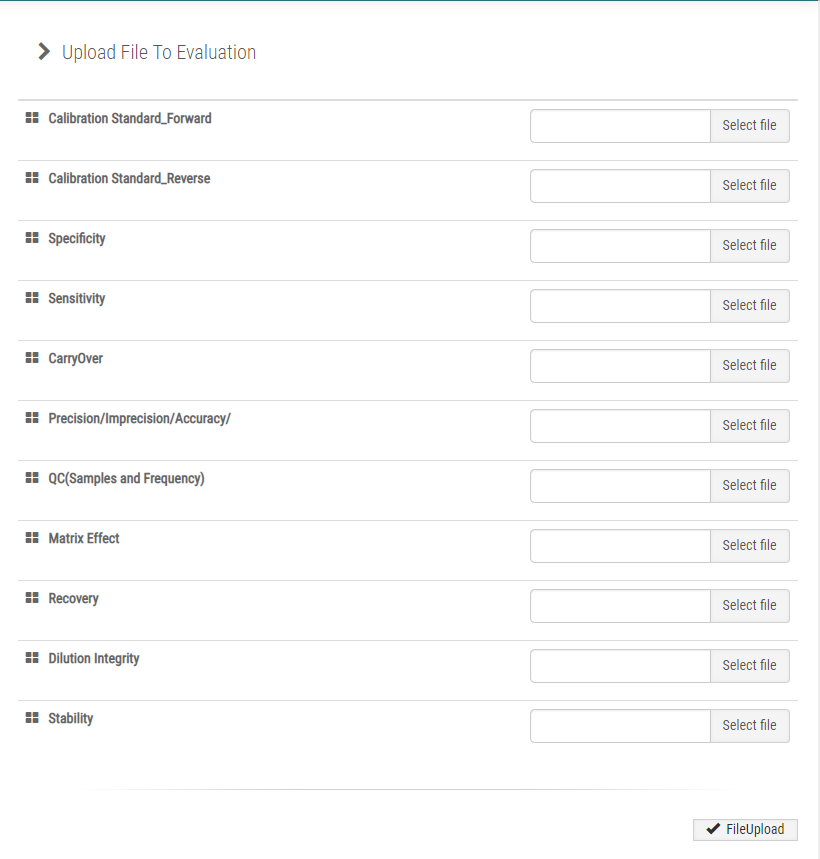


Supplementary Figure S 16

**Step 5: Expected Concentration Upload**

Users must insert theoretical concentration into the .csv files corresponding to **Calibration Standard**, **QC**, and **Dilution Integrity** that are uploaded. When theoretical concentrations for the **Calibration Standard** are inserted, the linearity of the calibration curve will be displayed in the side menu under **features linear regression.**

• On the side menu, click **True Point: Reverse Calibration, True Point: QC, and True Point: Dilution Integrity** under the **Data tab.**

• For **True Point: Calibration Curve**, insert all **Light/Heavy Concentrations**.

• For **True Point: QC,** insert all QC concentration points.

• For **True Point: Dilution Integrity,** insert initial dilution concentration point.

• Click **Submit.**

• The page will be redirected to a blank upload page if the upload was successful.

For the AFP-L3 experiment, the linear range contains eight concentration points, ranging from 0.0512 to 4000.0 ng/mL. The midpoint, the concentration of the internal standard, is set at the middle of the linear range, which is 19.2 ng/mL. For Reverse Calibration Curve, **Light Concentration** represents the internal standard, whereas **Heavy Concentration** represents various points within the range, and vice versa for the Forward Calibration Curve. The portal automatically generates boxes based on the values from the uploaded calibration file. The user fills in values for all points, including decimals, and clicks **Submit**. A successful entry will refresh the webpage. An example of inserting theoretical points for a calibration curve is shown below.


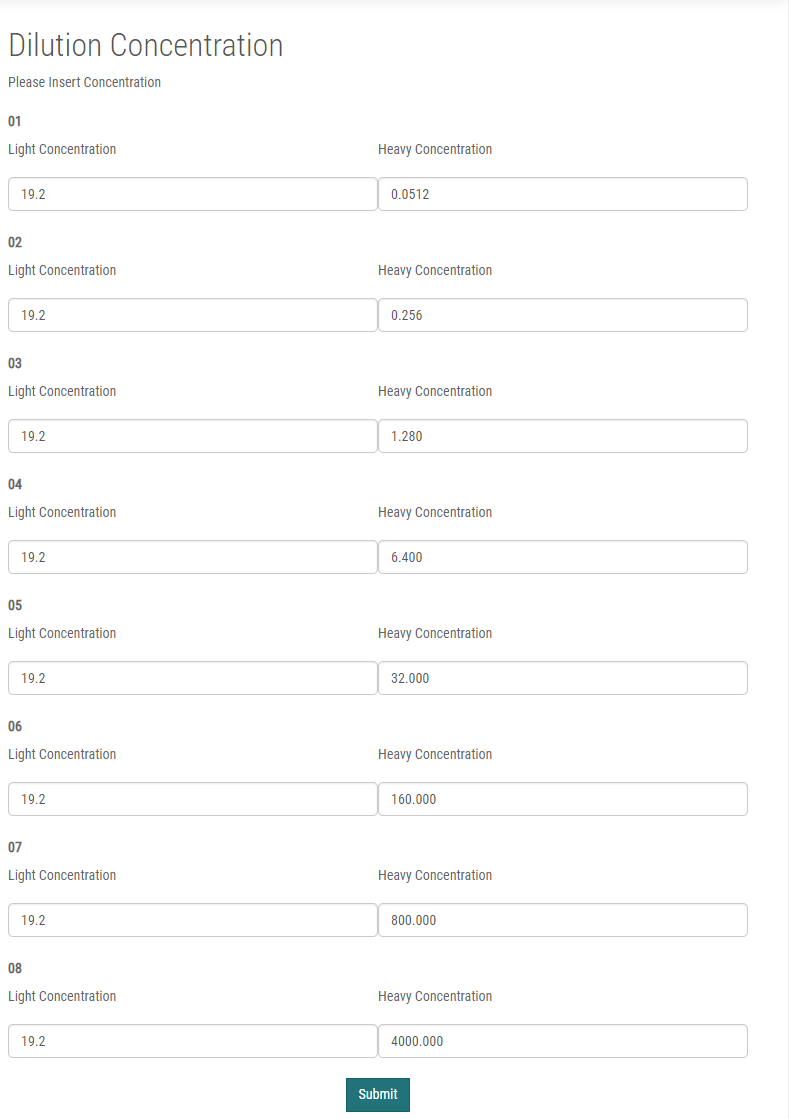


Supplementary Figure S 17

The page will redirect to a blank upload page if upload was successful.


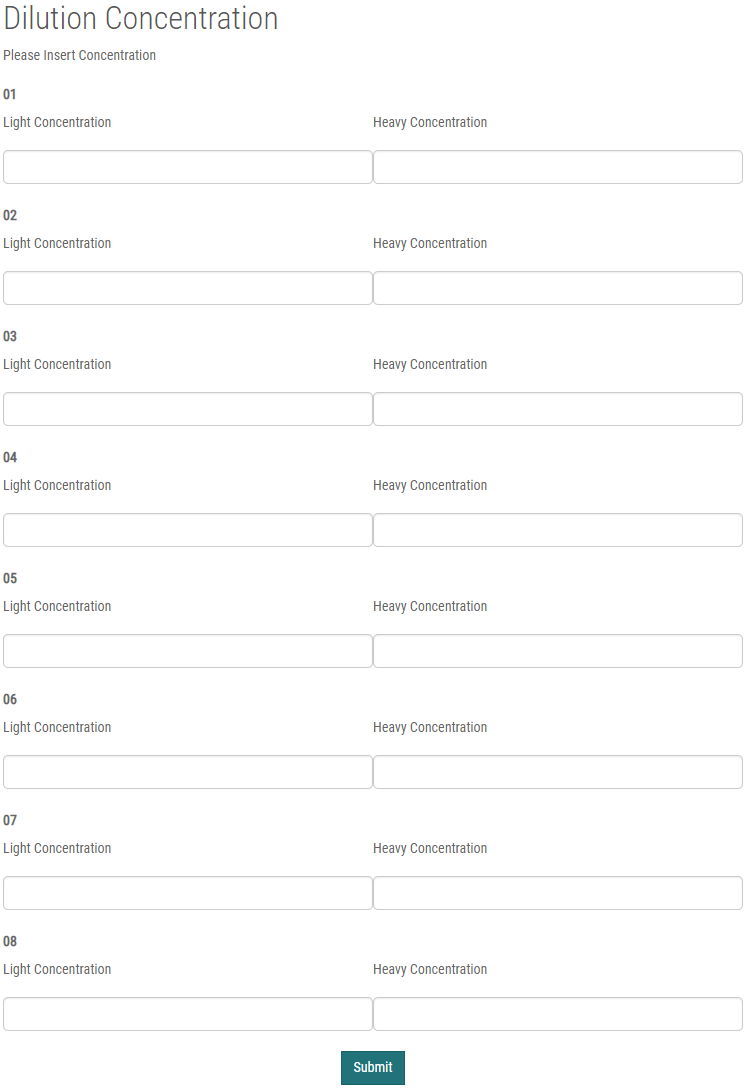


Supplementary Figure S 18

The process for inputting theoretical QC points is nearly identical to the previous step. The AFP-L3 experiment has QC points of 0.0512, 0.1540, 2000.0, and 4000.0 ng/mL. Insert the appropriate values and click **Submit**. Across all experiments light peptide is spiked with same concentration. Thus, **True Point: Calibration Curve** provides the internal standard concentration to the portal.


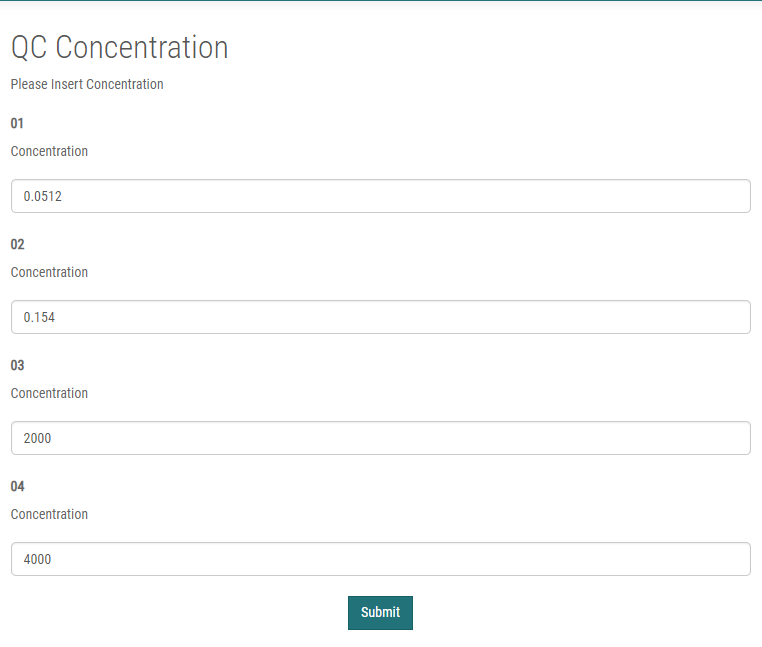


Supplementary Figure S 19

When successfully submitted, the page will revert to its original state.


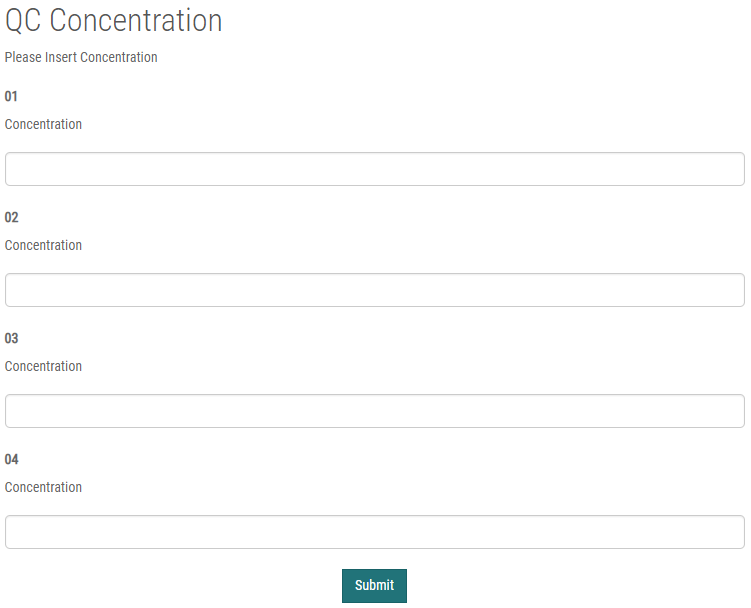


Supplementary Figure S 20

For Dilution Integrity, the AFP-L3 experiment used an initial concentration of 8000.0 ng/mL. Insert the corresponding value of the user’s experiment and click the **Submit** button. The internal standard is tested at the same concentration across all experiments; thus, the portal gets the internal standard concentration from **True Point: Calibration Curve**.


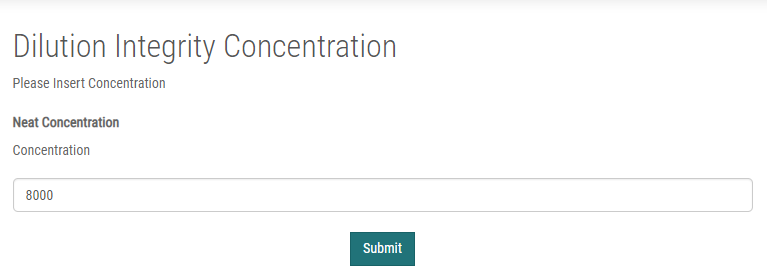


Supplementary Figure S 21

When successfully submitted, the page will refresh and change to the original blank state.


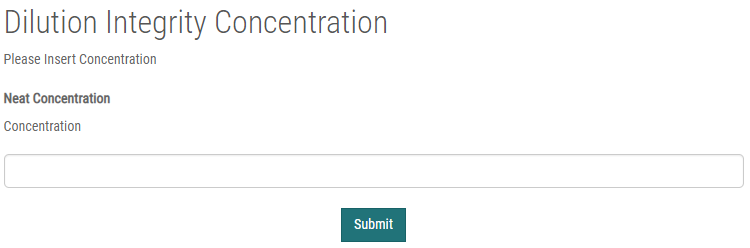


Supplementary Figure S 22

**Step 6: Linear Equation Selection**

In the Linear Regression page, the user can check the linearity of the calibration curve. Data from the uploaded csv file and the theoretical concentration are used for calculations. The user can check the slope, intercept, and R square of every transition with normal, Log_2_, or Log_10_ scales on the calibration curve. Once the user determines which scale will be used for calculations, the user can lock the scale by examining the regression result with the same scale.

• On the side menu, click **Feature Linear Regression** on **Data tab.**

• Choose **Normal/Log_2_/Log_10_** to select the scale of the calibration curve.

• Click **Select.**

• The page will refresh and display the regression result with the desired scale.

• Click **Slope, Intercept,** and **R square** to change the order of the table.


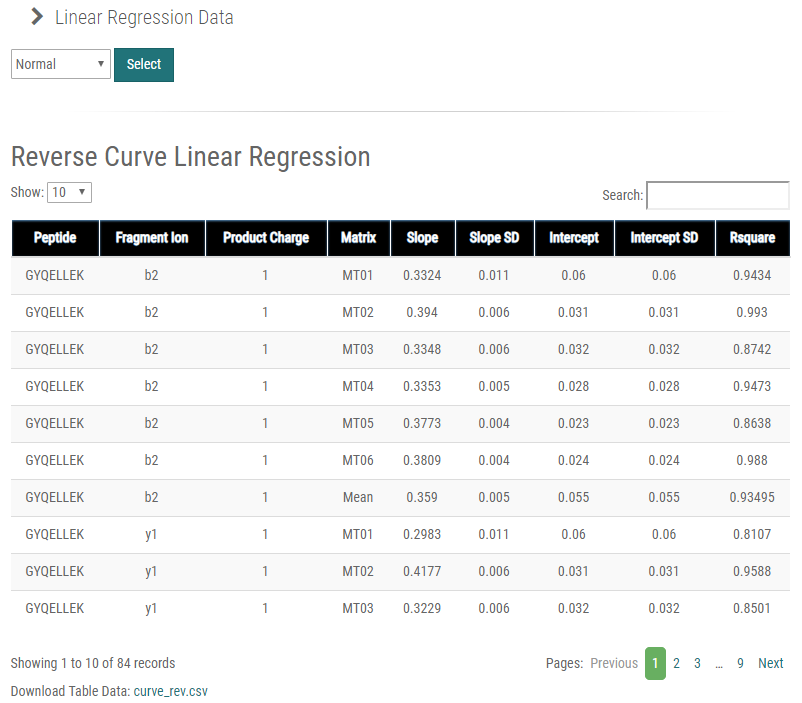


Supplementary Figure S 23

The most recently selected scale is used in subsequent calculations. There is no limitation to the number of target entries. M-MVP will process the given information and display the results in a table, which is available for download.


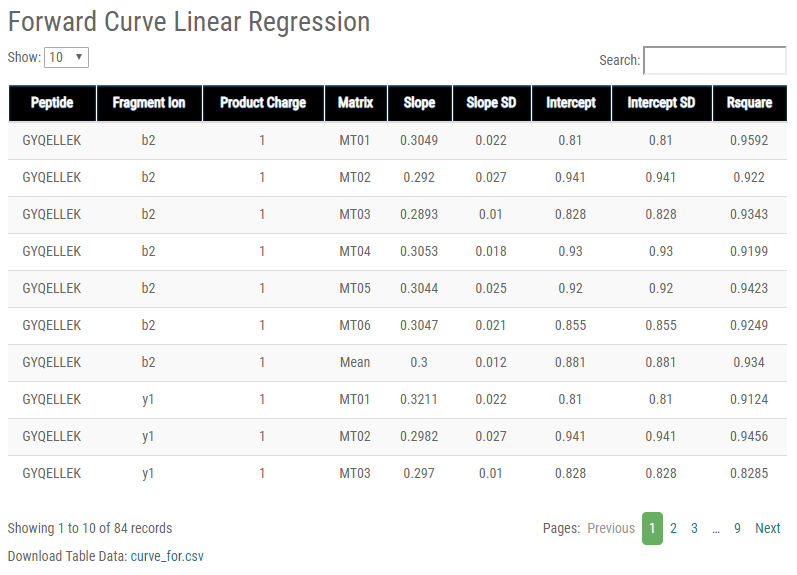


Supplementary Figure S 24

The Matrix column refers to sample matrices used in the experiment; for example, DB for double blank (matrix only) and CB for curve blank (matrix and internal standard). As mentioned above, the most recently selected scale is used for subsequent calculations. Click on the option box, which will display Normal as default. Select Log_10_ and wait for the webpage to refresh. Although the box will still display Normal, the Log_10_ scale will have been applied to the linearity results. The result will provide information regarding the linearity equations for each matrix. The combination of **Peptide**, **Fragment Ion**, and **Product Charge** that will be used is selected by the user, based on the information provided in the table.


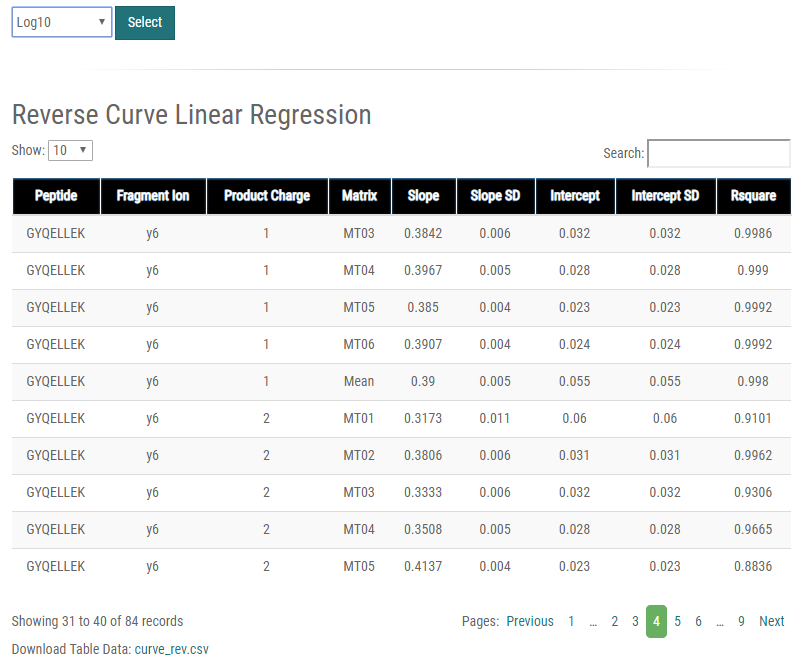


Supplementary Figure S 25

In this example, the concentration of the GYQELLEK peptide is calculated using a slope of 0.390 and intercept of 0.055, with an R^2^ value of 0.998 on log_10_ scale. After completion of the aforementioned steps, the calculations and results of the evaluation can be accessed by the user from the top and side menu.

**Step 7: Calculation Result Pages**

All 11 categories have their own results page. For each category, the user can check and download the calculated results in table form. In all results tables, **Protein** **Name**, **Peptide** **sequence**, **Fragment** **Ion**, and **Product** **Charge** are always displayed, whereas **Matrix** and **Calibrator** are displayed only when applicable. Various calculated values are shown depends on the category.

• Click **Reverse/Forward Calibration Curve, Specificity, Sensitivity, Carryover, Precision & Accuracy, Matrix Effect, Recovery, Dilution Integrity, Stability,** and **QC samples and frequency** under results in the side menu in the **Calculation tab** to see results.

• Click on **Columns (ex: Average, CV etc.)** to change the order of the table.

• Click on **Download** to download the data table as a csv file.

**(1) Reverse/Forward Calibration Curve**

The **Calibration Standards** tab displays both Reverse and Forward Calibration data. Given that the reverse calibration curve is the main function used for concentration calculations, its data are shown first. Users can examine **PAR**, **Standard Deviation,** and **CV** of peak area ratio in the uploaded csv file. Additionally, bias and signal-to-ratio values of reverse curves are shown. Results of the forward calibration curve are shown below, along with **PAR**, **Standard Deviation,** and **CV** of peak area ratio. For this tutorial, the y6 fragment ions of the GYQELLEK and VDFTEIQK peptides are used for quantification of the target protein. The **Average PAR**, **Standard Deviation**, and **CV** are obtained by averaging the mean results of the three replicates for a given matrix and calibrator. All experimental procedures were replicated as dictated by the administrations guideline.


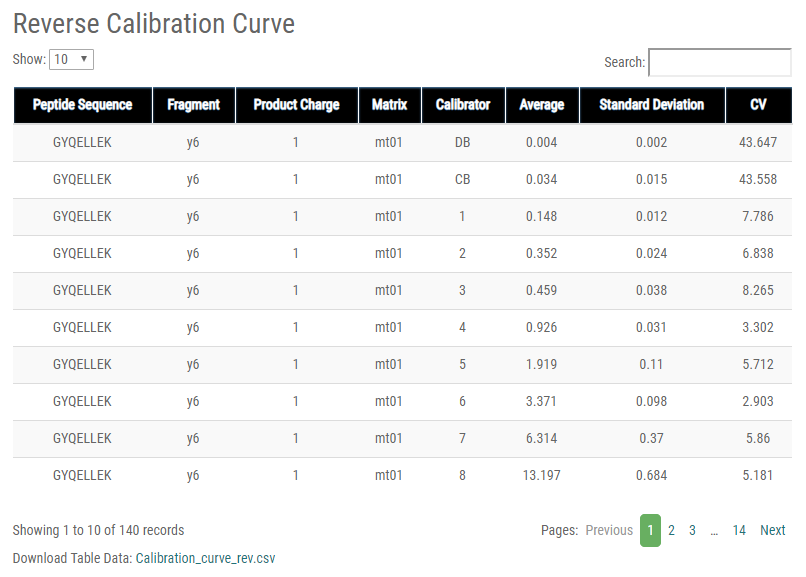


Supplementary Figure S 26


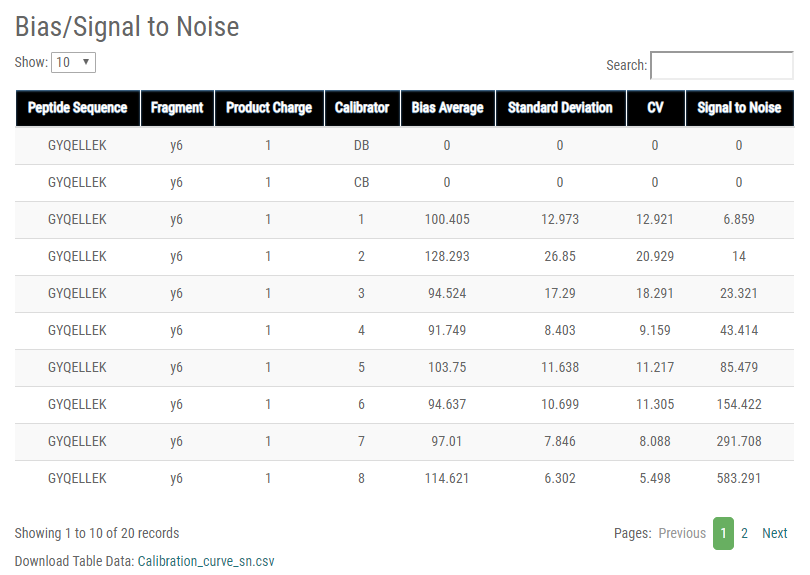


Supplementary Figure S 27


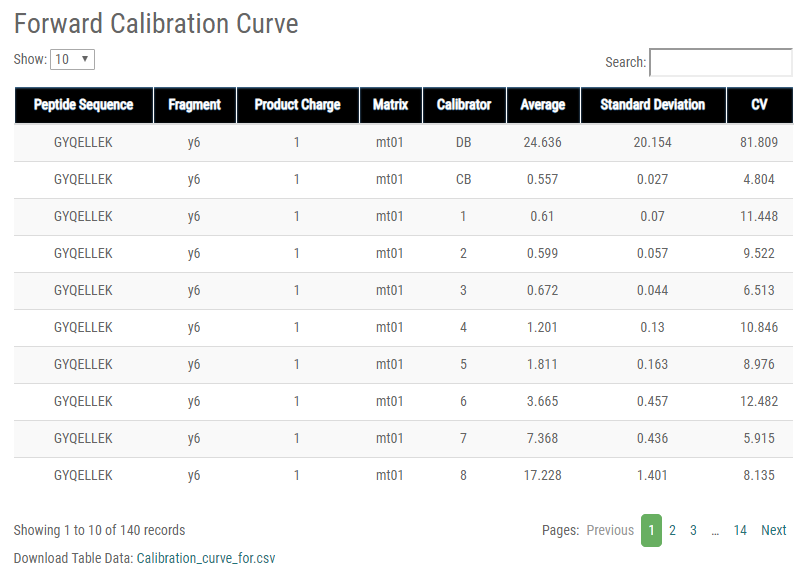


Supplementary Figure S 28

**(2) Specificity**

The **Specificity** tab displays **Average, Standard Deviation**, **CV,** and **Specificity** values of the peak area in the uploaded csv file. Specificity is calculated by dividing the double blank area by the LLOQ peak area and multiplying by 100.


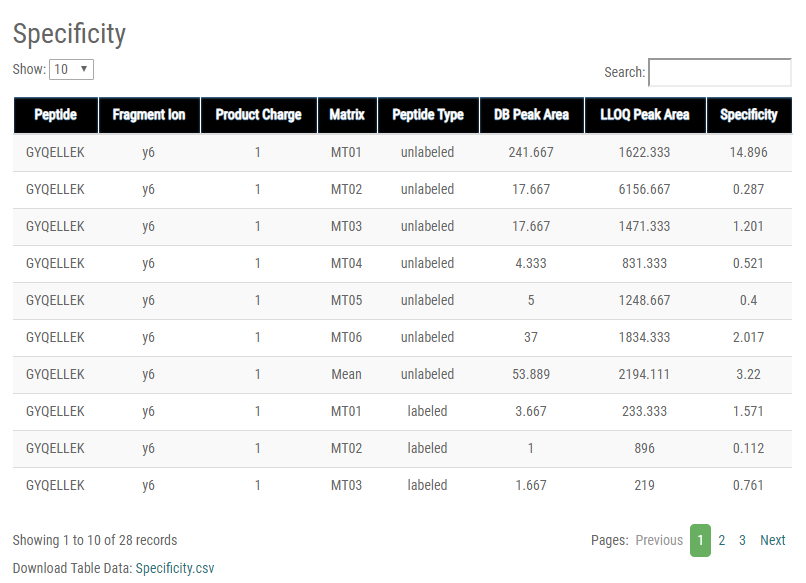


Supplementary Figure S 29

**(3) Sensitivity**

The **Sensitivity** tab shows **Average, Standard Deviation**, **CV (Precision), Signal to Noise (S/N),** and **Accuracy** values of the peak area ratio in the uploaded csv file. PAR of LLOQ and curve blank (CB) values are used to calculate S/N, dividing PAR_LLOQ_ by PAR_CB_. Concentration values are used to calculate accuracy by dividing the LLOQ concentration by the concentration of CB.


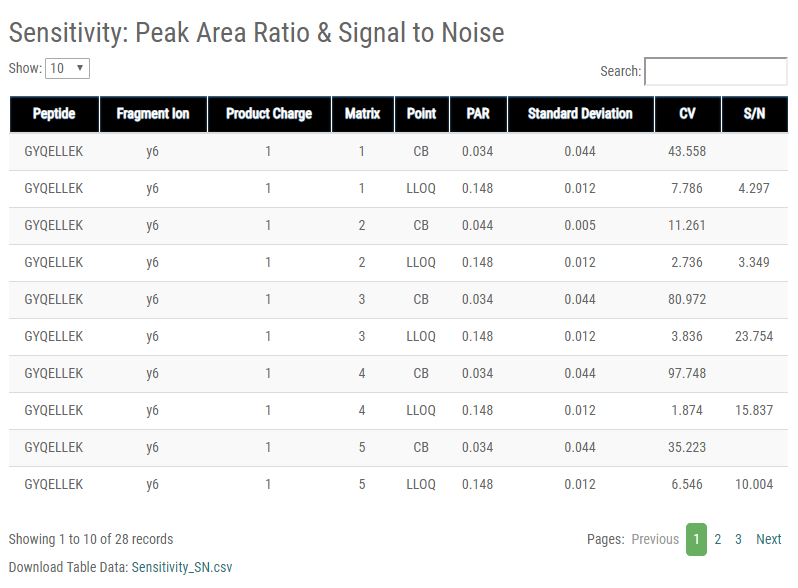


Supplementary Figure S 30


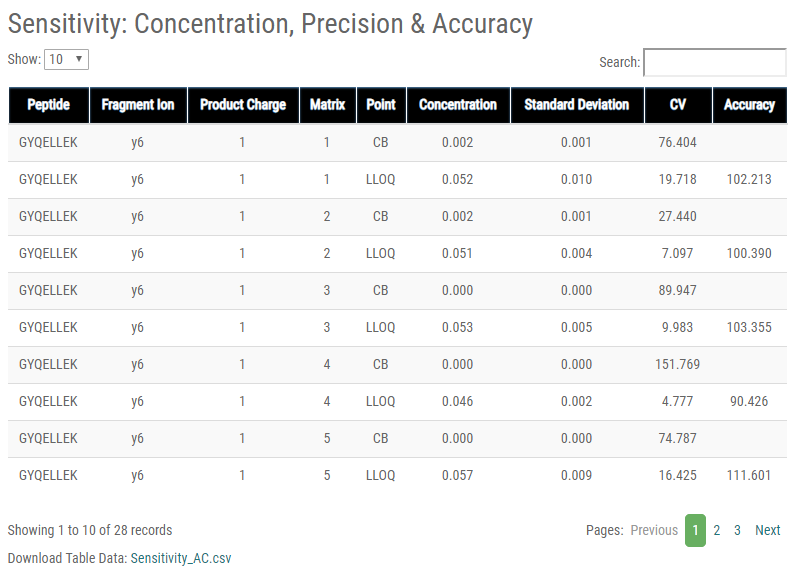


Supplementary Figure S 31

**(4) Carryover**

The **Carryover** tab displays **Average, Standard Deviation,** and **CV** values of the peak area in the uploaded csv file. Carryover is assessed by dividing the double blank after the ULOQ peak area with the LLOQ peak area and multiplying by 100.


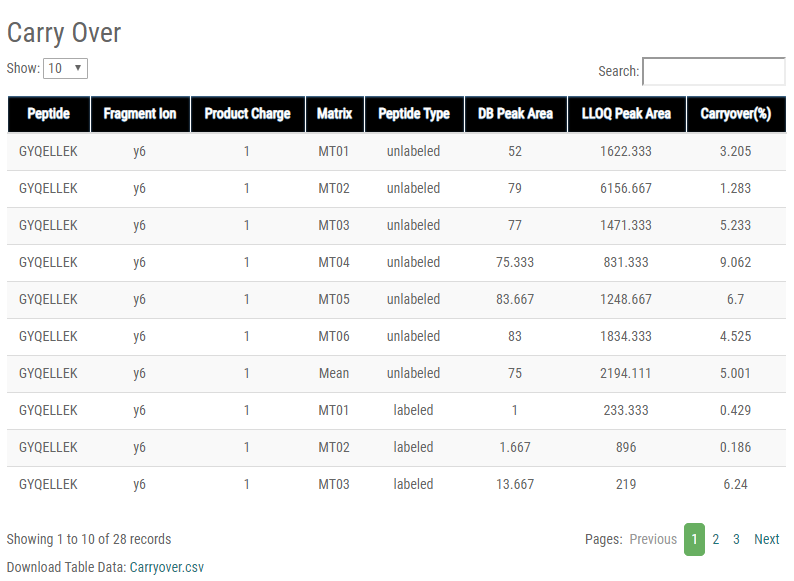


Supplementary Figure S 32

**(5) Precision and (6) Accuracy**

In the uploaded csv file, the **Precision and Accuracy** tab contains **Average, Standard Deviation,** and **CV** values of the peak area ratio. For Intra-Assay, the CV values and concentrations of each replicate on a given day were calculated and averaged. For Inter-Assay, the first replicate of each day was averaged to calculate between-run precision and accuracy. In the third table, mean values of Intra- and Inter-Assay CV are shown, with Total CV calculated as CV = 
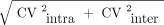
.


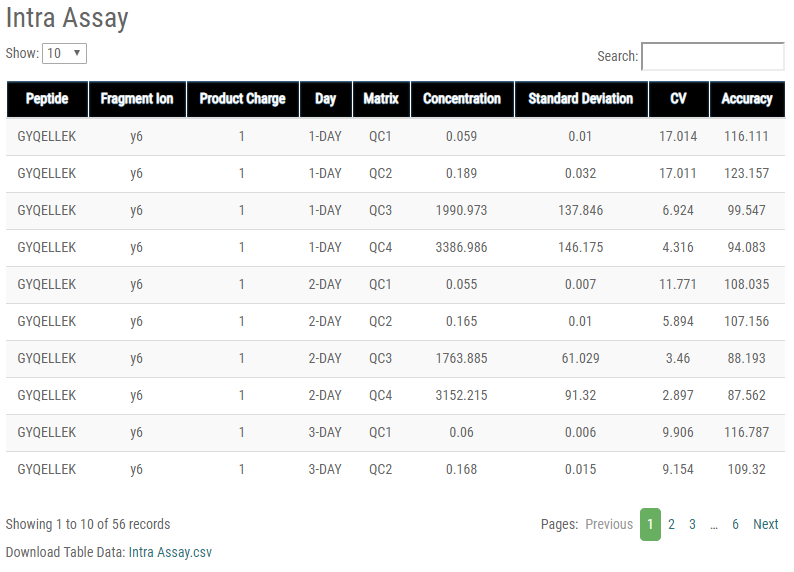


Supplementary Figure S 33


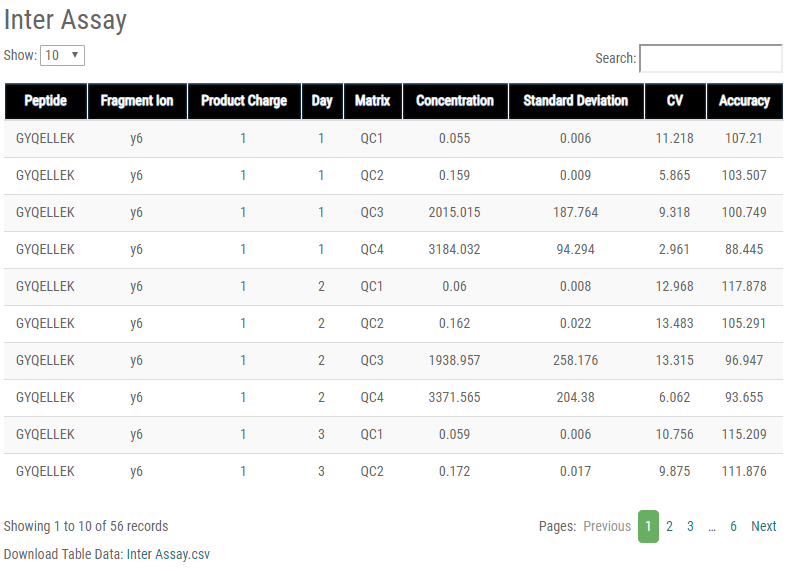


Supplementary Figure S 34


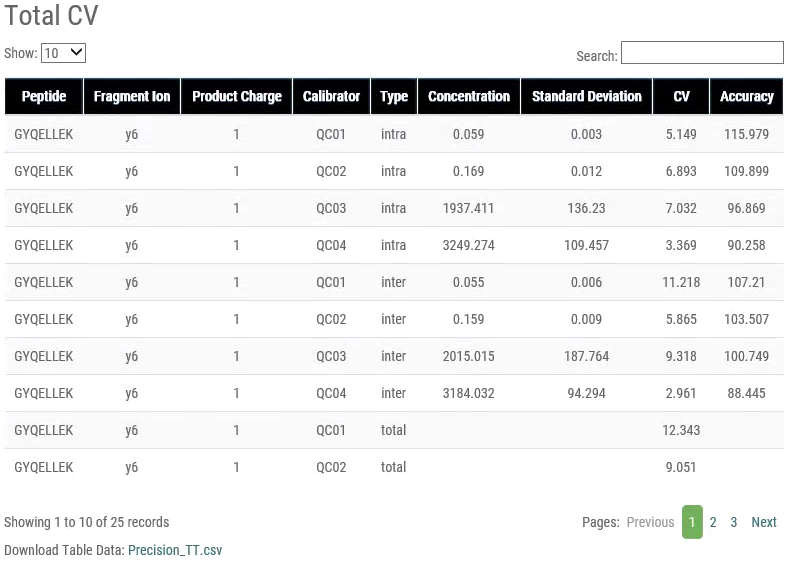


Supplementary Figure S 35

**(7) Matrix Effect**

In the uploaded csv file, the **Matrix Effect** tab contains **Average, Standard Deviation,** and **CV** values of the peak area ratio. Matrix effects are calculated by comparing the peak area ratios of the analyte in serum matrix versus in neat solution at the same concentration. The resulting ratio was expressed as %matrix effect.


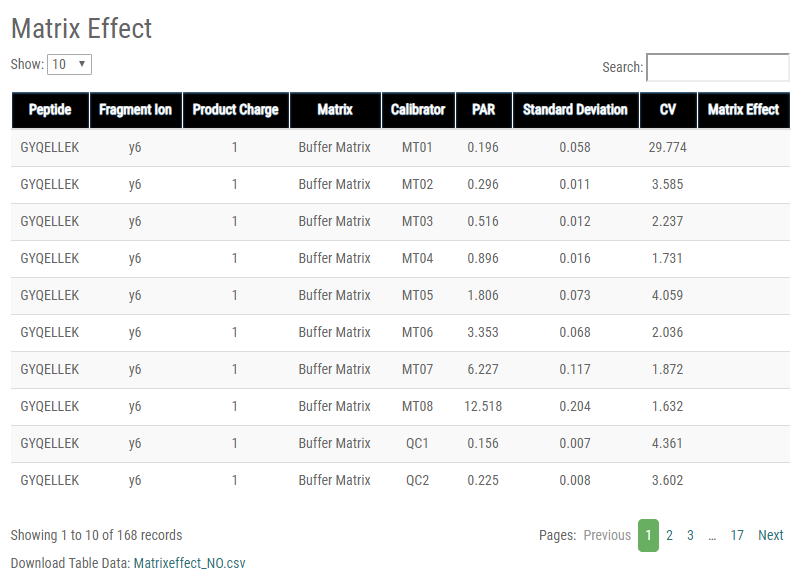


Supplementary Figure S 36


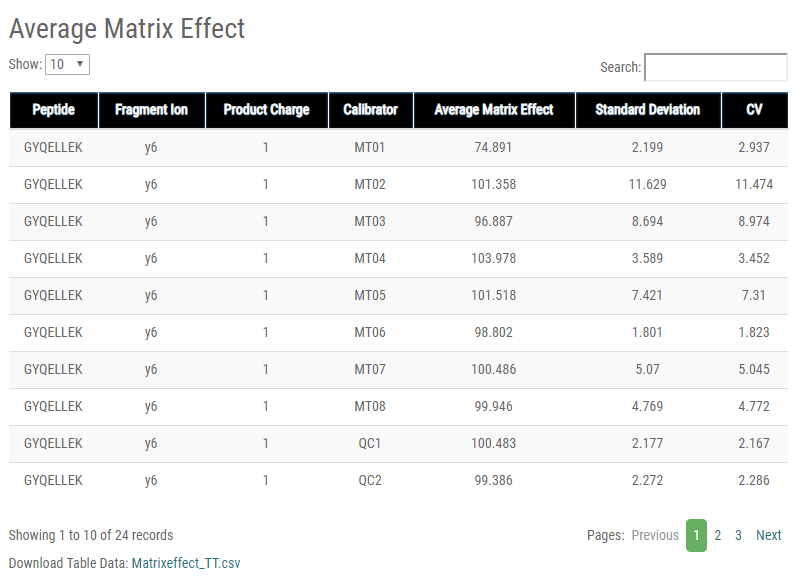


Supplementary Figure S 37

**(8) Recovery**

In the uploaded csv file, The **Recovery** tab contains **Average, Standard Deviation,** and **CV** values of the peak area ratio. Recovery is expressed as the relative peak area ratio recovery of enriched versus unenriched samples.


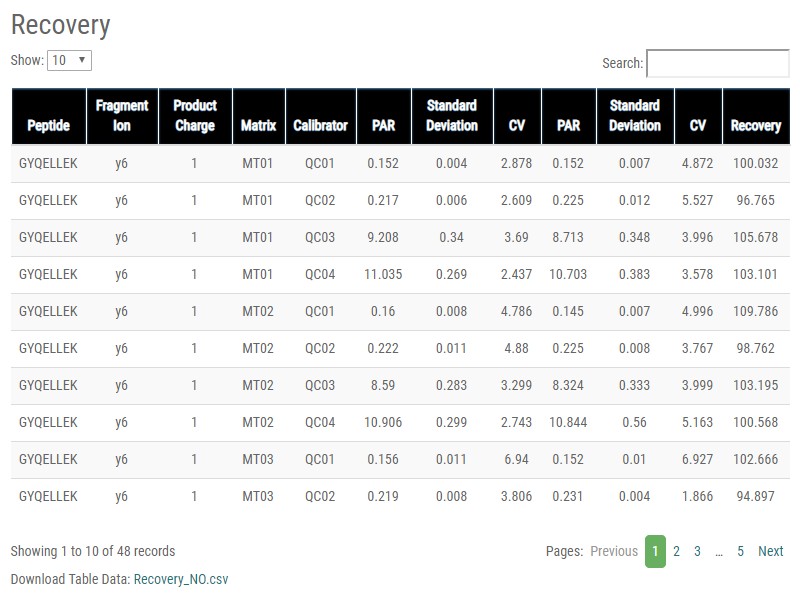


Supplementary Figure S 38


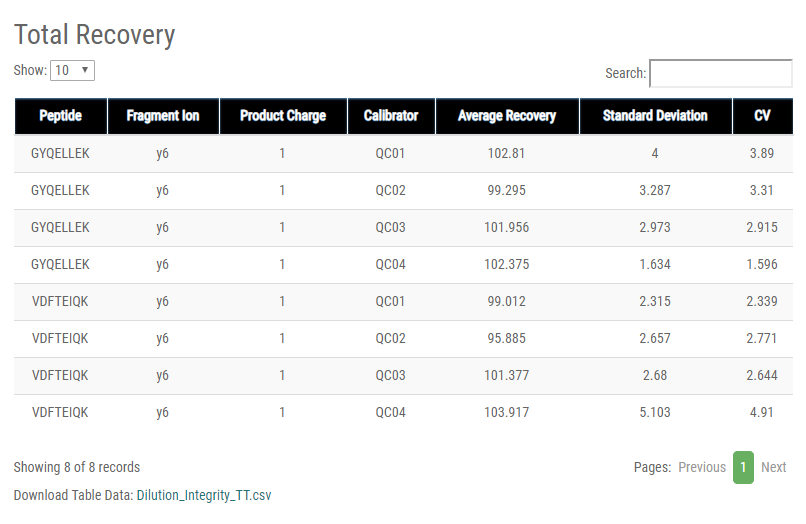


Supplementary Figure S 39

**(9) Dilution Integrity**

In the uploaded csv file, **the Dilution Integrity** tab contains **Average, Standard Deviation, CV, Dilution Concentration,** and **Percent Change** of concentration. Dilution factors in replicate names are used to convert the measured concentration to the diluted concentration, allowing the portal to compare changes with actual dilution concentrations.


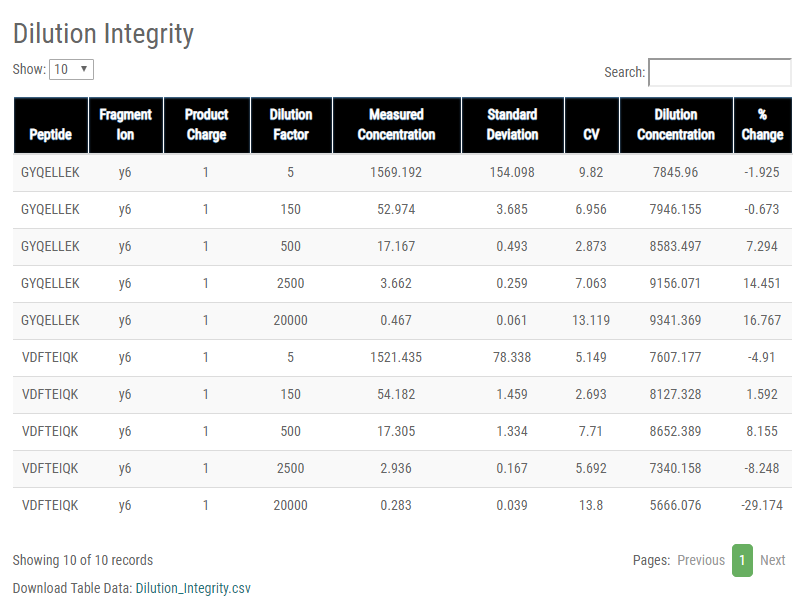


Supplementary Figure S 40

**(10) Stability**

In the uploaded csv file, the **Stability** tab contains **Average, Standard Deviation,** and **CV** values of the peak area ratio. Stability at each condition is calculated as the mean difference from the baseline value (0 days or 0 cycles).


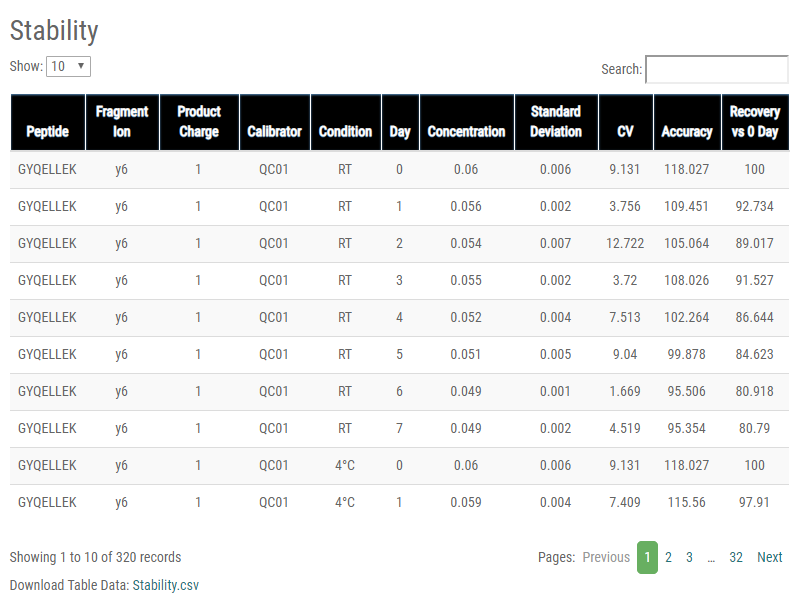


Supplementary Figure S 41

**(11) QC samples and frequency**

In the uploaded csv file, the **QC** tab contains **Accuracy** values of peak area ratio. For every measured QC concentration, the measured concentration is divided by the expected concentration and multiplied by 100.


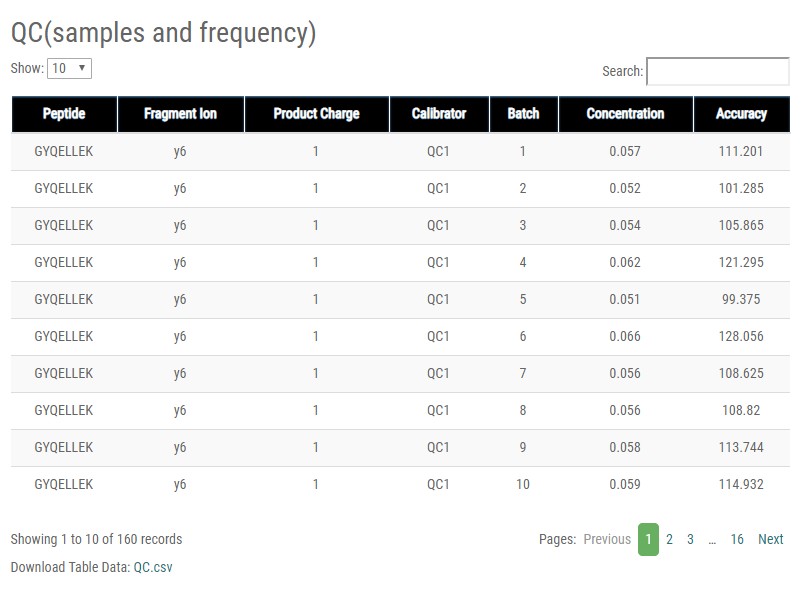


Supplementary Figure S 42

**Step 8: Evaluation Result Page**

The key function of M-MVP, the method validation results, is shown in the **Method Evaluation tab** on the top and side menu. Only those categories uploaded in the csv file are shown. Possible results include pass, not pass, or not addressed, depending on the calculated results. The result shown as “Pass” means that the guideline indicates strict criteria for both the experiment (validation practices) and result (performance specification)—that the M-MVP can automatically compare criteria to the calculated results from the uploaded data. Data that do not pass the standard are shown as “Not passed.” Some categories have no specific standard or are not mentioned in the guidelines; they are shown as “Not Addressed.”

• Click **Method Evaluation** in the side menu.

For the AFP-L3 experiment, we uploaded all method validation categories. The results show that all categories passed the guideline standard.


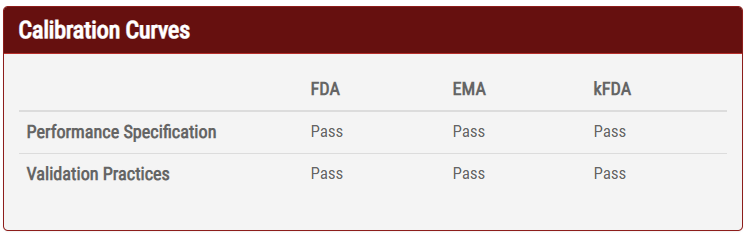

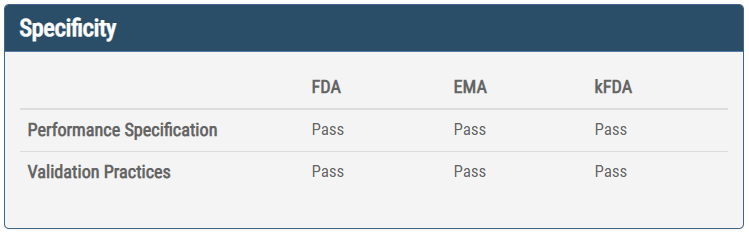

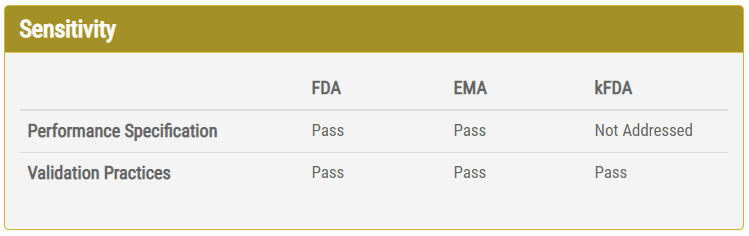

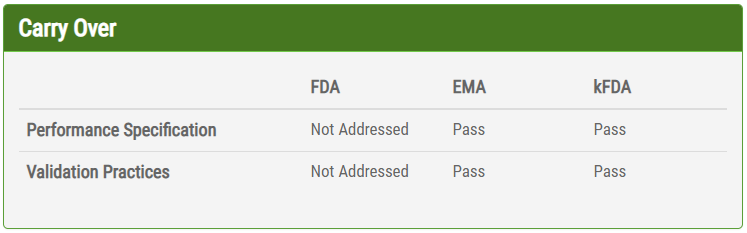

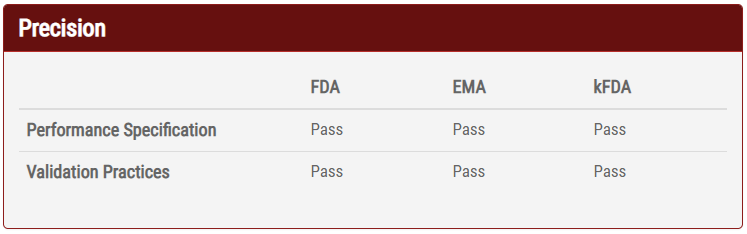

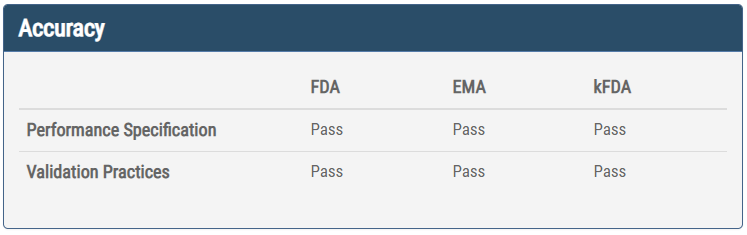

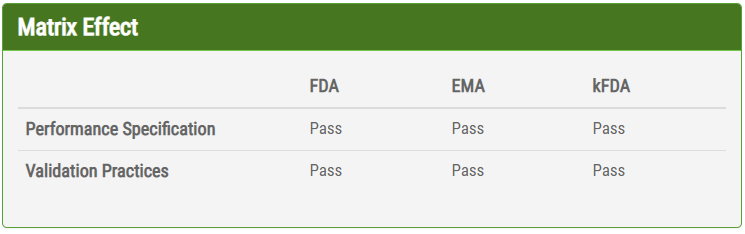

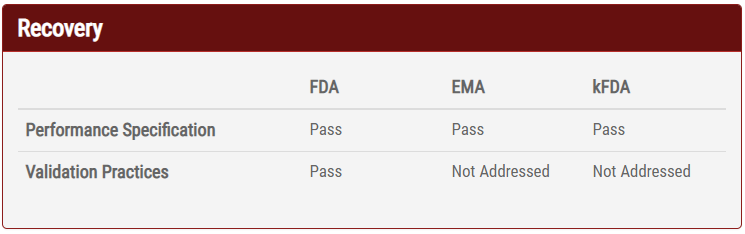

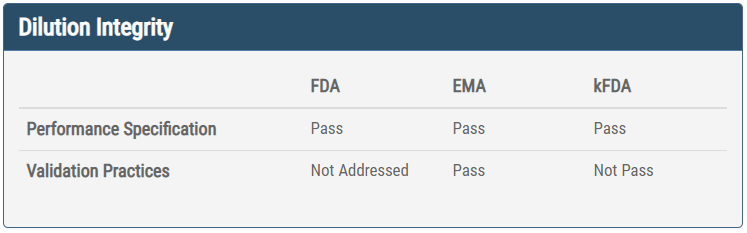

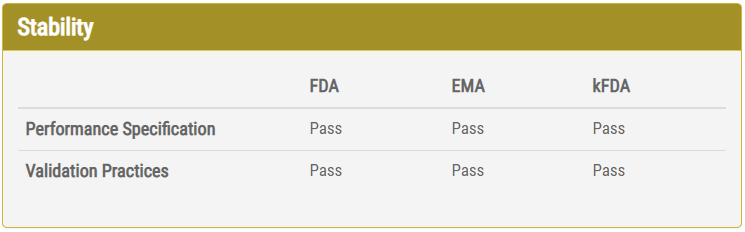


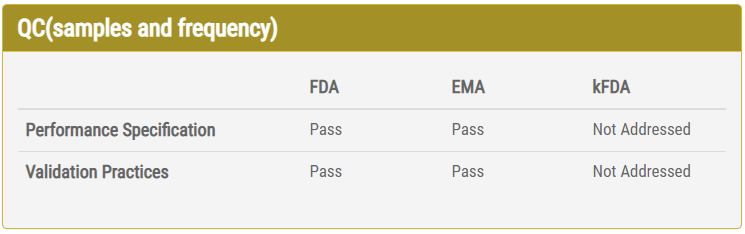


Supplementary Figure S 43

In this tutorial, every category passed the validation standard of all three regulatory agencies, except for Dilution Integrity.


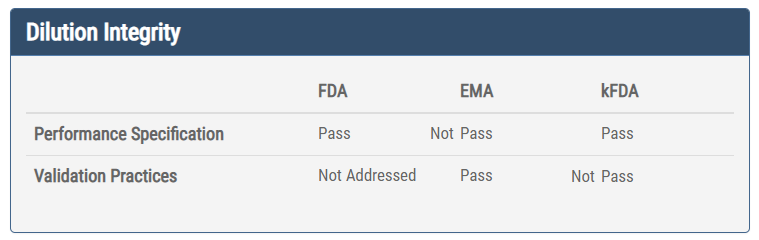


Supplementary Figure S 44

**Step 9: Re-Validation Process**

The user may experience validation failures, such as Matrix Effect CV values greater than 20%. In this situation, the user will have to conduct a new experiment or modify the Skyline data and re-export to a csv file. The following steps will guide the user through the re-validation process.

• Move the mouse cursor to **Validation,** then **Data** and click **Experimental Information.**

For tutorial, **“20170425_10250421”** is selected for the AFP-L3 experiment from the validation process mentioned above. The same experiment ID is used for further instruction.

• Select **“20170425_10250421.”**


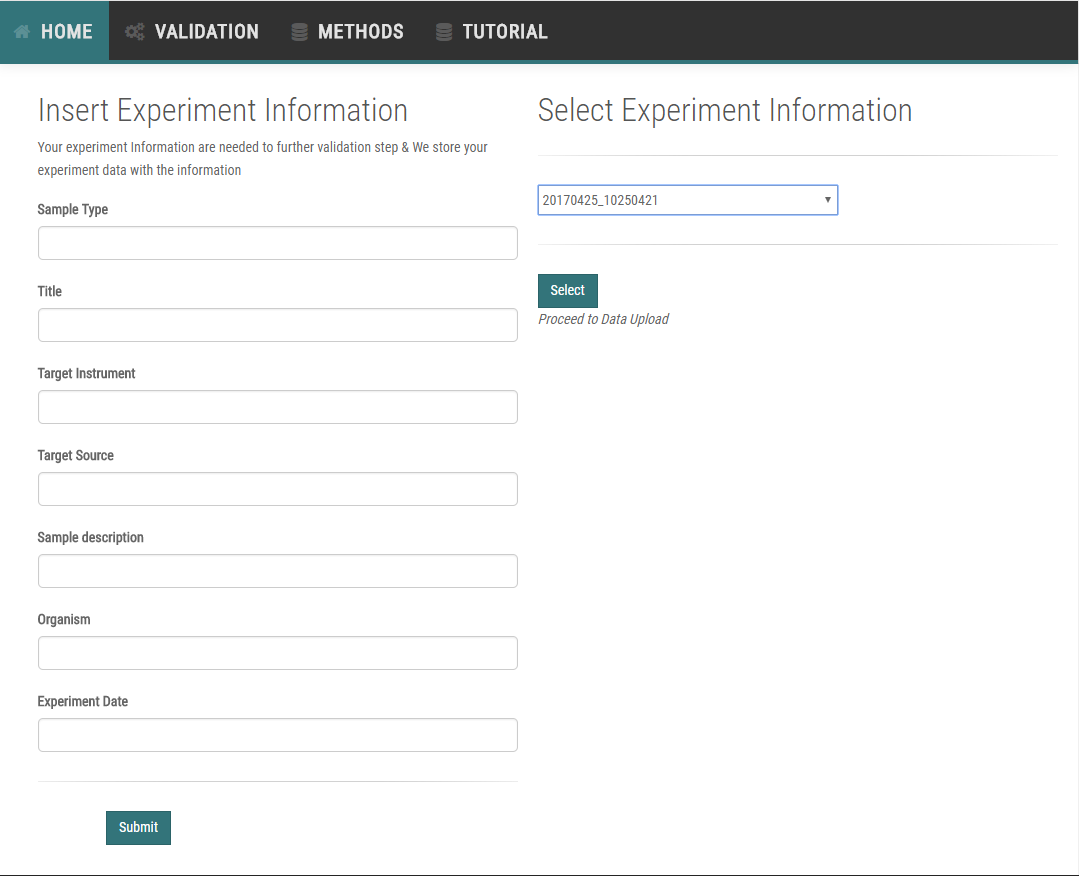


Supplementary Figure S 45

**(1) File upload**

As described in **Step 3-1**, each time a new file is uploaded for any category, M-MVP will discard the previous uploaded data and use the newly uploaded file for a new calculation. In this case, we will re-upload a **Dilution Integrity** csv file for re-validation.

• Click **Select File** of **Dilution Integrity** that the user is trying to upload as a Skyline-generated .csv file.

• Choose the file and click OK.

• Click **FileUpload.**

• The page will turn to a blank upload page if successfully uploaded.


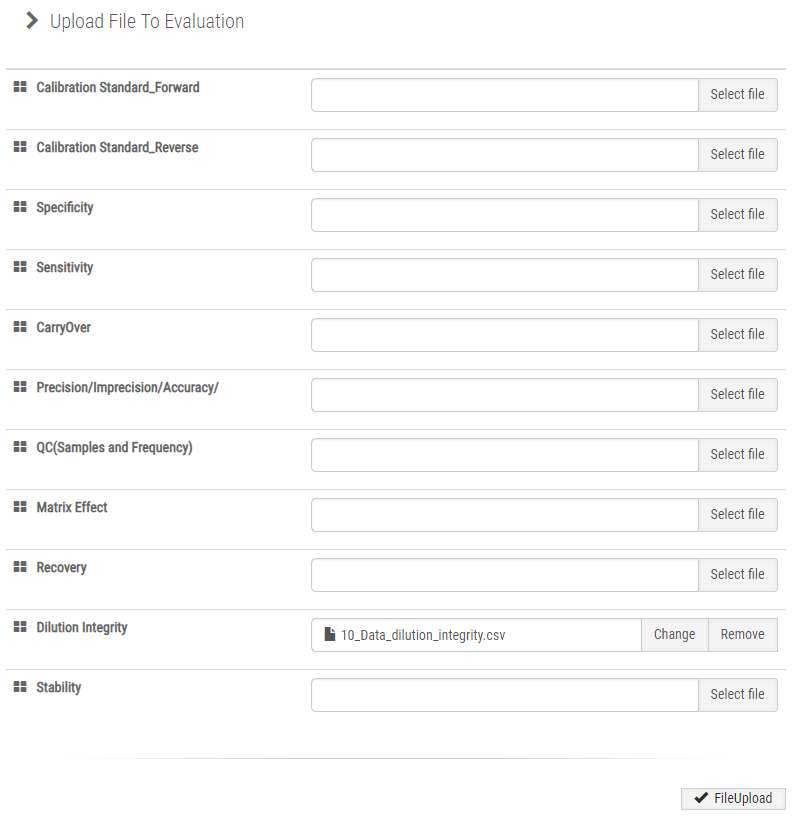


Supplementary Figure S 46

As described above, when the data are uploaded, the **dilution integrity** data from the previous upload will be discarded.

**(2) Theoretical concentration**

In addition to re-uploading Dilution Integrity data, we also have to re-insert theoretical concentrations for Dilution Integrity. In this case:

• On the side menu, click **Dilution Integrity Point** in the **Data tab**

• For **Dilution Integrity Point,** insert the initial dilution concentration point.

• Click **Submit.**

For Dilution Integrity, the AFP-L3 experiment used an initial concentration of 8000.0 ng/mL. Insert this value and click **Submit** button.


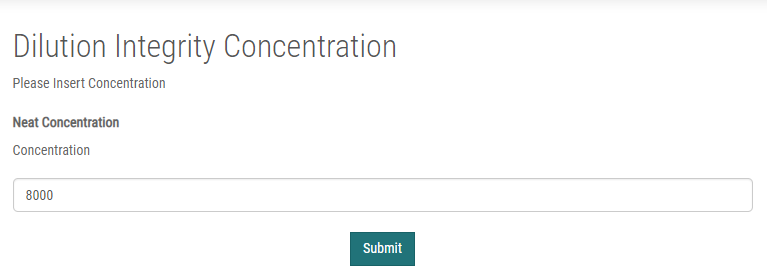


Supplementary Figure S 47

**(3) Calculation result**

In any **re-upload process,** the user must check the calculation output of the categories that were re-uploaded. In this case, we will click on the **Dilution Integrity page** under the **Calculation** tab:


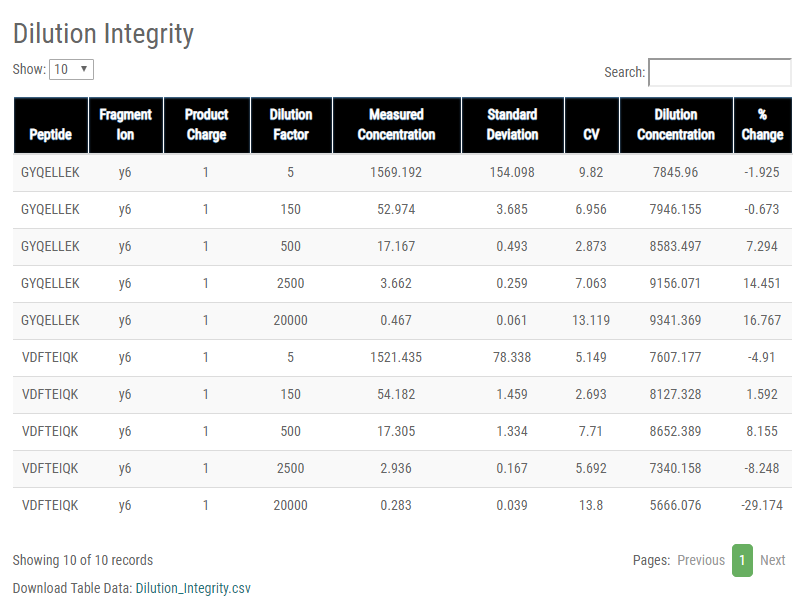


Supplementary Figure S 48

The **Dilution Integrity** tab contains **Average, Standard Deviation, CV, Dilution Concentration,** and **Percent Change** of the peak area ratio in the newly uploaded csv file, including any changes to the data made by the user.

**(4) Evaluation result**

After checking the re-uploaded categories, the evaluation results will be refreshed with the most recent data. Although only dilution integrity was re-uploaded, the results of other categories are still intact:

• Click **Method Evaluation** in the side menu.

As shown in the evaluation results page, the **Dilution Integrity** category passed the validation.


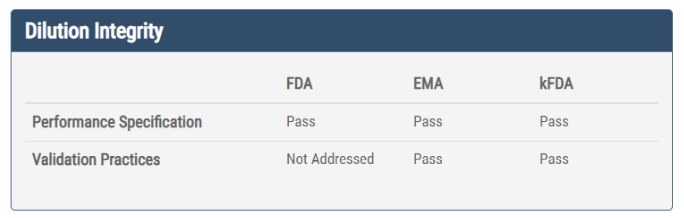


Supplementary Figure S 49
